# ^19^F-centred NMR analysis of mono-fluorinated compounds†

**DOI:** 10.1039/d1ra08046f

**Published:** 2022-03-30

**Authors:** Alan J. R. Smith, Richard York, Dušan Uhrín, Nicholle G. A. Bell

**Affiliations:** EaStCHEM School of Chemistry, University of Edinburgh David Brewster Rd Edinburgh EH9 3FJ UK Nicholle.Bell@ed.ac.uk

## Abstract

Addressing limitations of the existing NMR techniques for the structure determination of mono-fluorinated compounds, we have developed methodology that uses ^19^F as the focal point of this process. The proposed ^19^F-centred NMR analysis consists of a complementary set of broadband, phase-sensitive NMR experiments that utilise the substantial sensitivity of ^19^F and its far reaching couplings with ^1^H and ^13^C to obtain a large number of NMR parameters. The assembled ^1^H, ^13^C and ^19^F chemical shifts, values of *J*_HF_, *J*_HH_, and *J*_FC_ coupling constants and the size of ^13^C induced ^19^F isotopic shifts constitute a rich source of information that enables structure elucidation of fluorinated moieties and even complete structures of molecules. Here we introduce the methodology, provide a detailed description of each NMR experiment and illustrate their interpretation using 3-fluoro-3-deoxy-d-glucose. This novel approach performs particularly well in the structure elucidation of fluorinated compounds embedded in complex mixtures, eliminating the need for compound separation or use of standards to confirm the structures. It represents a major contribution towards the analysis of fluorinated agrochemicals and (radio)pharmaceuticals at any point during their lifetime, including preparation, use, biotransformation and biodegradation in the environment. The developed methodology can also assist with the investigations of the stability of fluoroorganics and their pharmacokinetics. Studies of reaction mechanisms using fluorinated molecules as convenient reporters of these processes, will also benefit.

## Abstract 2

^19^F is the focal point of broadband, phase-sensitive 2D NMR experiments that provide ^1^H, ^13^C and ^19^F chemical shifts, values of *J*_HF_, *J*_HH_, and *J*_FC_ coupling constants and ^13^C-induced ^19^F isotopic shifts to elucidate structures of fluorinated molecules.

## Graphical abstract

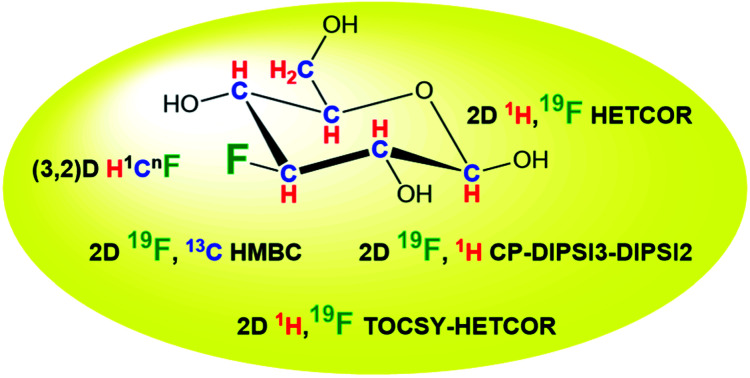

## Introduction

Fluorine's unique properties, such as high electronegativity, strength of a single fluorine–carbon bond and small atomic radius, impart significant benefits to fluorinated organic molecules.^1^ Fluorination has been shown to enhance potency and/or specificity of molecular interactions, increase membrane permeability, modulate metabolism, moderate the p*K*_a_ of proximal functionalities, influence conformation, stabilise inherently reactive functionalities and produce viable bioisosteres.^2,3^ Currently, about 20% of the commercial pharmaceuticals contain fluorine and the proportion of newly approved fluoro-pharmaceuticals is rising steadily.^4,5^ The proportion of fluoro-agrochemicals is even larger; 53% of all active agrochemicals registered during 1998–2020 belong to this category.^6^ Similarly, ^18^F is the most frequently used radioisotope in positron emission tomography radiopharmaceuticals.^7^ Fluorination also has the potential to become a useful tool for improving properties of fragrance and semiochemical molecules.^8^

To capitalise on the ability of fluorine to improve molecular properties, there is a drive to design efficient and environmentally-safe chemical,^9,10^ enzymatic^11^ and chemo-enzymatic^12–14^ fluorination methods. To assist these efforts, efficient analytical methods for the characterisation of fluorinated molecules are required. ^19^F NMR spectroscopy plays a prominent role in this area due to the favourable properties of ^19^F, such as its high sensitivity, 100% natural abundance, large chemical shift dispersion, large and far-reaching spin–spin interactions and ^13^C induced ^19^F isotopic shifts.

The lack of background ^19^F signals, due to the scarcity of fluorinated endogenous compounds, makes ^19^F NMR perfect for the analysis of mixtures produced by chemical or chemoenzymatic reactions with minimum clean-up steps or compound separation required. 1D ^19^F NMR spectroscopy has been widely used in studies of biodegradation and biotransformation of fluorinated compounds^15–21^ mostly relying on the use of known standards^15^ or tabulated ^19^F chemical shifts. In a similar manner, ^19^F NMR has also been used for probing the mechanism and kinetics of chemical reactions, were fluorine is a convenient reporter of the processes taking place.^22,23^

In support of such wide ranging activities, we have developed a ^19^F-centred NMR approach for the analysis of mono-fluorinated compounds, taking ^19^F NMR beyond recording simple 1D NMR spectra. Put together, the information obtained allows the structure elucidation of fluorine-containing molecular moieties and complete structure determination of small fluorine-containing molecules. It is well suited for the studies of complex mixtures. The ^19^F-centred NMR shares similarities to the “NMR spy” approach developed for the analysis of complex mixtures of soil organic matter, where –O^13^CH_3_ tags are introduced to a subset of molecules.^24–26^ Nevertheless, there are significant differences between the two approaches. Firstly, fluorinated molecules already contain ^19^F and therefore do not require additional chemical modifications. Secondly, the fluorine atom is typically closer to the protons and carbons of an organic molecule than are the nuclei of the –O^13^CH_3_ group which, when combined with far reaching ^19^F couplings, allows to inspect parts of the molecule that are more remote from the ^19^F “tag.” The FESTA family of NMR experiments^27–29^ that relies on selective manipulation of individual ^1^H and ^19^F resonances illustrated this approach and provided ^1^H–^19^F chemical shift correlations and ^1^H–^19^F coupling constants when such spin manipulations were possible.

Our methodology utilises the far reaching ^1^H–^19^F and ^19^F–^13^C couplings to obtain ^1^H and ^13^C chemical shifts of nuclei multiple bonds away from the ^19^F atom, provides accurate values of numerous *J*_HF_, *J*_FC_, and *J*_HH_ coupling constants and ^13^C induced ^19^F isotopic shifts from several purposely designed nonselective 2D NMR experiments. Their advantages over similar existing NMR experiments are highlighted. The ^19^F-centered approach is illustrated using 3-fluoro-3-deoxy-d-glucose, 1, which can be characterized as a simple mixture of two ^19^F-containing molecules. Application of this methodology to a very complex mixture of compounds produced by chloramination of a single fluorinated molecule is presented elsewhere.^30^

## Experimental

The sample of 3-fluoro-3-deoxy-d-glucose (30 mg), 1, was dissolved in 600 μL of D_2_O (Merck, 99.9 atom% D) and placed into a 5 mm NMR tube. Spectra involving ^19^F were acquired at 300 K on a 400 MHz Bruker Avance III NMR spectrometers equipped with a TBO BB-H/F-D probe. A 1D ^1^H spectrum was acquired on an 800 MHz Bruker Avance III NMR spectrometer equipped with a TCI 5 mm probe. Parameters of the performed NMR experiments are presented in Table S1† and Bruker pulse sequences compatible with TopSpin 3 can be found in the ESI† (pp. 1–6).

**Fig. 1:**
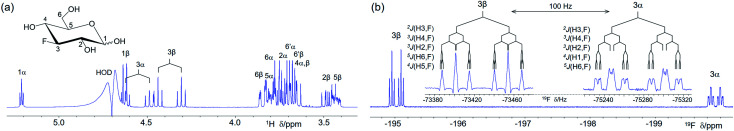
(a) 400 MHz 1D ^1^H spectrum of 1 (structure in the inset) with HOD suppression and resonance assignments; (b) ^1^H-coupled 1D ^19^F spectrum of 1. Expansions of ^19^F multiplets of both anomeric forms from resolution-enhanced spectra produced using Lorentzian to Gaussian line shape conversion (LB = −2.0 Hz, GB = 0.5) are given in the inset.

**Fig. 2:**
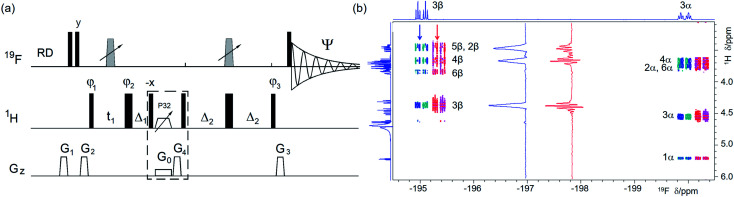
(a) Pulse sequence of a ^19^F-detected z-filtered 2D ^1^H, ^19^F HETCOR. In a non z-filtered experiment, the part within the dashed rectangle is not included. For explanation of symbols used for pulses see Experimental. The NMR parameters used are given in Table S1.† The delays used were as follows: *Δ*_1_ = *p*44; *Δ*_2_ = one half of the *J*_HF_ evolution; *t*_1_(0), the initial *t*_1_ evolution delay time = 0.5 × in0, where in0 is the *t*_1_ increment. The gradient strengths were as follows: *G*_0_ = 3%; *G*_1_ = 17%; *G*_2_ = 31%; *G*_3_ = 24%; *G*_4_ = 10.0%. The following phase cycling was used: *φ*_1_ = *x*, −*x*; *φ*_2_ = 4*x*, 4(−*x*); *φ*_3_ = 2*y*, 2(−*y*); *Ψ* = *x*, 2(−*x*), *x*. States-TPPI protocol was used for sign discrimination in *F*_1_ with the phase *φ*_1_ incremented by 90°. Purging of ^19^F magnetisation at the beginning of the pulse sequence by a composite 90° ^19^F pulse and pulsed field gradients (PFGs) minimises the cancellation artefacts. (b) An overlay of the ^19^F-detected 2D ^1^H, ^19^F HETCOR spectra with (blue/turquoise) and without the z-filter (red/magenta). For clarity, the spectrum acquired without a z-filter was offset horizontally to the right. Insets show 1D *F*_1_ traces taken at positions indicated by arrows. 1D ^1^H and ^19^F spectra are shown along the left and top, respectively.

**Fig. 3:**
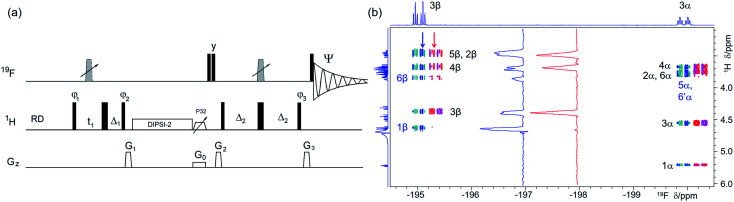
(a) Pulse sequence of a 2D ^1^H, ^19^F TOCSY–HETCOR. For explanation of symbols used for pulses see Experimental. The NMR parameters used are given in Table S1.†The delays were as follows: *Δ*_1_ = *p*44; *Δ*_2_ = one half of the *J*_HF_ evolution; *t*_1_(0) is the initial *t*_1_ evolution delay time = 0.5 × in0, where in0 is the *t*_1_ increment. The gradient strengths were are follows: *G*_0_ = 5%; *G*_1_ = 17%; *G*_2_ = 31%; *G*_3_ = 24%. The following phase cycling was used: *φ*_1_ = *x*, −*x*; *φ*_2_ = 4*x*, 4(−*x*); *φ*_3_ = 2*y*, 2(−*y*); *Ψ* = *x*, 2(−*x*), *x*, −*x*, 2*x*, −*x*. States-TPPI protocol was used for sign discrimination in *F*_1_ with the phase *φ*_1_ incremented by 90°. Purging of ^19^F magnetisation after the z-filter by a composite 90° ^19^F pulse followed by the *G*_2_ PFG minimises the cancellation artefacts. (b) An overlay of the ^19^F-detected 2D ^1^H,^19^F TOCSY–HETCOR spectrum (blue/turquoise) and a z-filtered VT ^19^F-detected 2D ^1^H, ^19^F HETCOR spectrum (red/magenta, horizontally offset to the right) of 1 acquired with the pulse sequence shown in (a) and Fig. 2a, respectively. Vertical traces of the two spectra as indicated by arrows are shown in the inset. Exclusive/stronger TOCSY cross peaks are labelled in blue. 1D ^1^H and ^19^F spectra are shown along the left and the top, respectively.

**Fig. 4:**
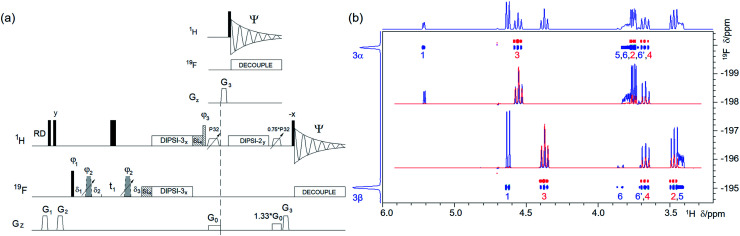
(a) Pulse sequence of a 2D ^19^F, ^1^H CP-DIPSI3–DIPSI2. For explanation of symbols used for pulses see Experimental. The NMR parameters used are given in Table S1.†The dashed line indicates signal acquisition before an optional ^1^H–^1^H spin-lock. For description of pulses see Experimental. The delays were as follows: *δ*_1_ = 20 μs; *δ*_2_ = *δ*_1_ + (2/π) × *p*3; *δ*_3_ = *p*2; *t*_1_(0) is the initial *t*_1_ evolution delay time = 0.5 × in0, where in0 is the *t*_1_ increment. The gradient strengths were as follows: *G*_0_ = 5%; *G*_1_ = 17%; *G*_2_ = 31%; *G*_3_ = 66%. The following phase cycling was used: *φ*_1_ = *y*, −*y*; *φ*_2_ = 4*x*, 4(−*x*); *φ*_3_ = 2*y*, 2(−*y*); *Ψ* = *x*, 2(−*x*), *x*. The states-TPPI protocol was used for sign discrimination in *F*_1_ with the phase *φ*_1_ incremented by 90°. Purging of ^19^F magnetisation at the beginning of the pulse sequence by a composite 90° ^19^F pulse and PFGs minimises the cancellation artefacts. (b) An overlay of two 2D ^19^F, ^1^H CP-DIPSI3–DIPSI2 spectra acquired with 20 ms ^19^F → ^1^H cross-polarisation (CP) only (red) and an additional 50 ms ^1^H → ^1^H spin-lock (blue) using the pulse sequence shown in (a). The red spectrum was offset vertically to facilitate visualisation of the cross peaks. The two insets show overlaid 1D F2 traces through ^19^F resonances of α- and β-d-glucose from both spectra. Twice as many scans were acquired for the blue spectrum as for the red spectrum. 1D ^19^F and ^1^H projections of the blue spectrum are shown along the left and top, respectively.

**Fig. 5:**
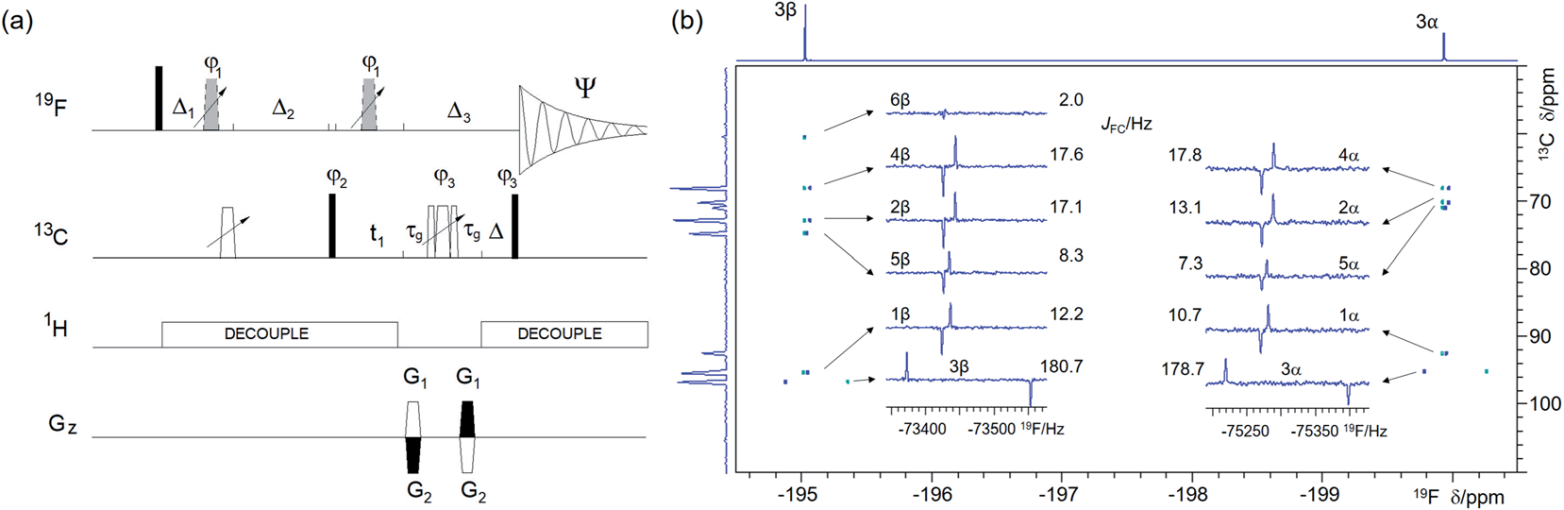
(a) Pulse sequence of the 2D ^19^F, ^13^C HMBC optimised for ^*n*^*J*_FC_ correlations. For explanation of symbols used for pulses see Experimental. The NMR parameters used are given in Table S1.† The delays were as follows: *d*6 = 0.25/^*n*^*J*_FC_; *Δ* = *p*44; *Δ*_3_ = 2 × *p*16 + 2 × *d*16 + *p*24 + *Δ* + 8 μs; *Δ*_1_ = *d*6 − *Δ*_3_/2; *Δ*_2_ = *d*6 + *Δ*_3_/2 − *p*14 + (2/π) × *p*1; *t*_1_(0) is the initial *t*_1_ evolution delay time = 0.5 × in0, where in0 is the *t*_1_ increment. The gradient strengths were are follows: *G*_1_ = 80%; *G*_2_ = cnst30 × *G*_1_, where cnst30 = (1 − sfo2/sfo1)/(1 + sfo2/sfo1) and sfo1 and sfo2 are ^19^F and ^13^C frequencies, respectively. The following phase cycling was used: *φ*_1_ = 2*x*, 2(−*x*); *φ*_2_ = *x*, −*x*; *φ*_3_ = 4*x*, 4(−*x*); *Ψ* = 2(*x*, −*x*), 2(−*x*, *x*). The echo-antiecho protocol was used with PFGs changing sign between real and imaginary increments. Phases *φ*_2_ and *Ψ* were incremented by 180° together with the PFG sign change, (b) A 2D ^19^F, ^13^C HMBC spectrum of 1 optimised for ^*n*^*J*_FC_ of 20 Hz acquired using the pulse sequence of shown in (a). The two insets show 1D *F*_2_ traces for individual ^13^C resonances of the α- and β-forms of 1. 1D ^1^H-decoupled ^19^F NMR spectrum and the ^13^C projection are shown on the top and along the left of the spectrum, respectively.

**Fig. 6:**
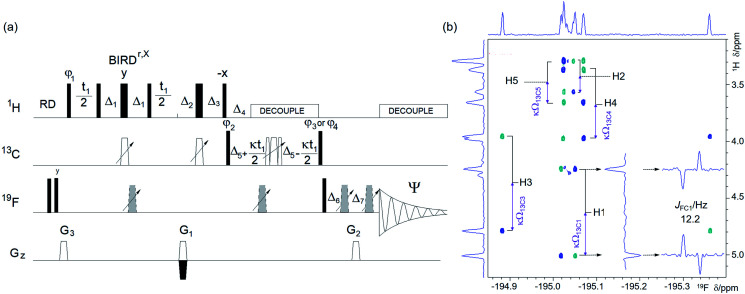
(a) Pulse sequence of the (3, 2)D H^1^C^*n*^F correlation experiment. For explanation of symbols used for pulses see Experimental. The NMR parameters used are given in Table S1.†The delays were as follows: *d*2 = 0.25/^1^*J*_HC_; *d*3 = 0.5/^1^*J*_HC_; *d*4 = 0.25/^*n*^*J*_FC_; *d*6 = cnst1/^1^*J*_HC_, where cnst1 = 0.5 for CH and 0.25 for CH_2_ groups; *Δ*_1_ = *d*3 − *p*14/2; *Δ*_2_ = *d*2 − *p*14/2 − *p*16 − *d*16; *Δ*_3_ = *d*2 − *p*14/2 − 2*t*_1_(0); *Δ*_4_ = *d*6; *Δ*_5_ = *d*4; *Δ*_6_ = *p*16 + *d*16 − (2/π)*p*1 + 4 μs; *Δ*_7_ = *p*16 + *d*16 + 4 μs, where *p*16 and *d*16 are the PFG length and the recovery time, respectively. The gradient strengths were as follows: *G*_1_ = 40%; *G*_2_ = 42.51%; *G*_3_ = 13%. The following phase cycling was used: *φ*_1_ = *y*, −*y*; *φ*_2_ = 4*x*, 4(−*x*); *φ*_3_ = 2*x*, 2(−*x*); *φ*_4_ = 2*y*, 2(−*y*); *Ψ* = *x*, 2(−*x*), *x*, −*x*, 2*x*, −*x*. The echo-antiecho protocol was used with *G*_1_ changing sign between real and imaginary increments. Phases *φ*_1_ and *Ψ* were incremented by 180° together with the sign change. Two interleaved experiments were acquired applying either the *φ*_3_ or *φ*_4_ phase to the last 90° ^13^C pulse, (b) an *F*_1_ antiphase (3, 2)D H^1^C^*n*^F spectra of 1 acquired using the pulse sequence shown in (a) showing the cross peaks of the β-anomer of 1. Positive and negative cross peaks are shown in blue and turquoise, respectively. The insets contain vertical and horizontal traces through the H1, F cross peaks. The ^1^H chemical shift of protons directly attached to ^13^C atoms and the associated *κΩ*_13C_ frequencies are indicated. Antiphase doublets in *F*_2_ show ^*n*^*J*_FC_ coupling constants. Horizontal and vertical internal projections are shown on the top and along the left side of all spectra, respectively. The editing process that simplifies this spectrum is explained in the text and shown in Fig. S5.†

The following symbols are used to depict the pulse sequences in Fig. 1–6: the thin and thick filled rectangles represent high power 90° (^1^H, *p*1 or ^19^F, *p*3) and 180° (^1^H, *p*2) pulses, respectively. 1 ms adiabatic CHIRP pulses with a peak power of 10.3 kHz (*p*44, shaded trapezoid with an inclined arrow) were applied to ^19^F. A 20 ms 60 kHz CHIRP ^1^H pulse with a peak power of 2294 Hz (*p*32, trapezoid with inclined arrow) was used as part of the z-filter. A 500 μs CHIRP pulse (*p*14) and 2 ms composite CHIRP pulse (*p*24) were applied to ^13^C with a peak power of 9800 Hz. Unless stated otherwise, the r.f. pulses were applied from the *x*-axis. The 100% pulsed field gradient strength corresponds to 53.5 G cm^−1^.

## Results and discussion

### 1D ^1^H and ^19^F spectra of 3-fluoro-3-deoxy-d-glucose, 1

A 400 MHz 1D ^1^H spectrum of 1 with the suppression of the HOD signal shows considerable overlap of ^1^H resonances (Fig. 1a). A ^1^H-coupled 1D ^19^F spectrum 1 (Fig. 1b) contains two ^19^F signals belonging to α- and β-anomeric forms of 1. The insets highlight numerous ^1^H–^19^F coupling constants of 1. NMR parameters of 1 obtained using the developed experiments, including those involving ^13^C, are presented in Table S2.†

### ^19^F-centred NMR experiments – novelty and hardware requirements

Although a number of NMR experiments exist that correlate ^19^F chemical shifts with those of other nuclei,^31^ the majority of existing techniques yield magnitude mode spectra.^32,33^ Acquisition of pure-phase absorption signals in a phase-sensitive manner is much preferred, as it provides higher sensitivity and allows for accurate determination of coupling constants, including identification of the active coupling constants. Some existing phase-sensitive experiments yield complicated cross peak structures that lower their sensitivity.^34,35^

The optimal performance of experiments constituting the ^19^F-centred NMR approach across a range of ^19^F frequencies, is ensured by the use of adiabatic inversion pulses.^36,37^ The experiments provide pure phase multiplets with simple structure afforded by ^1^H or ^19^F decoupling and were designed to minimise the effect of passive spins; they do not use refocusing intervals, which maximises their sensitivity. NMR hardware capable of pulsing simultaneously on ^1^H and ^19^F frequencies is required; fortunately, such systems are more widespread now. To access the rich information provided by ^13^C–^19^F interactions, a three-channel NMR spectrometer is necessary. Maximum benefits are realised on systems equipped with highly sensitive low temperature probes. These have also become more widely available, mainly due to their use in binding studies of biomacromolecules with fluorinated ligands.

### Fluorine–proton and proton–proton correlation

Following the acquisition of ^1^H-decoupled and ^1^H-coupled 1D ^19^F spectra, mapping of the ^1^H–^19^F correlations is the natural next step in investigating the structure of fluorinated compounds. For this task a choice of three types of experiment exist: hetero-COSY, HETCOR or HMBC.^31,32^ Most of these can be implemented using ^19^F or ^1^H as the directly detected nucleus. Using ^19^F as the directly detected nucleus, the 2D ^1^H, ^19^F HMBC has the highest sensitivity, but yields mixed-phased multiplets. 2D ^1^H, ^19^F hetero-COSY can be implemented with either nucleus being sampled in the directly detected (*F*_2_) dimension. Nevertheless, sampling ^19^F in the *F*_2_ dimension has a distinct advantage of acquiring spectra with the high digital resolution required for the identification of active and passive *J*_HF_ coupling constants and potentially also for their measurements. A disadvantage of COSY type spectra is the antiphase nature of their cross peaks (particularly in *F*_1_) and their large footprint.

Choosing to obtain the ^1^H–^19^F correlations using a phase-sensitive ^19^F-detected 2D ^1^H, ^19^F HETCOR experiment (Fig. 2a) retains the advantages of ^19^F detection. Its uniform performance across a large ^19^F chemical shift range is guaranteed by the use of broadband inversion CHIRP pulses^38^ arranged in a double inversion adiabatic sweep (Fig. S1†), a feature applied in several experiments presented here to eliminate phase evolution of the transverse magnetisation during pulses.^39–41^ This allows the use of such pulses not only for spin inversion but also refocusing.

The structure of cross peaks in HETCOR spectra is simplified by the application of a 180° ^19^F pulse in the middle of the *t*_1_ interval, reducing the probability of signal overlap in spectra of complex mixtures. A drawback of this experiment is the evolution of ^1^H–^1^H couplings during the defocusing interval 2*Δ*_2_, which competes with the evolution of ^1^H–^19^F couplings, decreasing its sensitivity. This decrease can often be tolerated because of the 100% natural abundance of both nuclei.

Due to diverse sizes of *J*_HF_ coupling constants, no attempt was made to refocus ^19^F magnetisation prior to detection and ^1^H decoupling was not applied during *t*_2_. Preserving the antiphase character of cross peaks is important, as it allows the identification of active couplings. Nevertheless, if a ^1^H-coupled ^19^F 1D spectrum is overlap free, it is advised to read the coupling constants from this spectrum, where accurate values are readily obtained (see Fig. 1b).

In a basic HETCOR experiment,^32^ the evolution of ^1^H–^1^H couplings during the ^1^H–^19^F defocusing interval, 2*Δ*_2_, leads to the appearance of mixed phase proton multiplets in *F*_1_ – a feature that is masked by the magnitude mode presentation of spectra. This issue was resolved in the proposed phase-sensitive experiment by inserting a z-filter^42^ after the *t*_1_ period, which separates the evolution of ^1^H–^1^H couplings during the *t*_1_ and the 2*Δ*_2_ defocusing interval. Providing the *t*_1max_ is kept short (<30 ms), the cross peaks appear as singlets in *F*_1_. The described features of the experiment are illustrated on a 2D ^1^H, ^19^F HETCOR spectrum of 1 (Fig. 2b), where correlations with many ^19^F coupled protons are observed.

Protons not coupled by a sizable (>1.0 Hz) coupling constant to a ^19^F, but which are part of a spin system containing at least one ^1^H coupled to a ^19^F, are detected in a 2D ^1^H, ^19^F TOCSY–HETCOR experiment (Fig. 3a). Here, the ^1^H chemical shifts are labelled before their magnetisation is spread through the network of *J*_HH_ coupled spins by a DIPSI-2 spin-lock.^43^ Part of the magnetisation that has reached the ^19^F-coupled protons is then transferred to ^19^F for detection in a subsequent HETCOR step. An overlay of the 2D ^1^H, ^19^F HETCOR and 2D ^1^H, ^19^F TOCSY–HETCOR spectra (Fig. 3b) revealed several protons with a *J*_HF_ close to zero, which were not detected by the HETCOR experiment. Other protons of both anomeric forms of 1 coupled with small coupling constants to ^19^F showed increased intensities.

In addition to *J*_HF_ coupling constants, *J*_HH_ coupling constants provide important structural information that for complex mixtures is inaccessible by standard 2D experiments, but can be retrieved when some form of ^19^F editing is used. In principle, ^1^H–^1^H couplings modulate cross peaks in the *F*_1_ dimension of the 2D (TOCSY–)HETCOR experiments discussed above but in practice, typical *t*_1_ acquisition times used to record such spectra are too short to resolve them. The ^1^H–^1^H couplings are more likely to be resolved in the *F*_2_ dimension of ^1^H-detected experiments considering a non-refocused 2D ^1^H-detected ^19^F, ^1^H HETCOR, this experiment shows *F*_2_ multiplets with *J*_HF_ and *J*_HH_ coupling constants as anti-phase and inphase splitting, respectively, complicating access to *J*_HH_ coupling constants (data not shown).

The *J*_HH_ coupling constants can be measured more effectively from inphase proton multiplets acquired in the presence of ^19^F decoupling. Developed for simple mixtures of fluorinated compounds, this reasoning has led to the design of FESTA experiments.^27–29^ These 1D selective experiments require that both ^19^F and ^1^H multiplets are amenable to selective inversion, which is rarely the case for complex mixtures; experiments that do not rely on selective manipulations of spins are more robust.

A suitable alternative involving the use of ^19^F → ^1^H cross-polarisation (CP) that produces inphase ^1^H multiplets was already proposed in the form of a 3D CP ^19^F, ^1^H heteronuclear TOCSY experiment.^44^ We did not find it necessary to label the ^1^H chemical shifts after the initial ^19^F → ^1^H magnetisation transfer and present here a 2D version of this experiment in the form of a 2D ^19^F, ^1^H CP-DIPSI3–DIPSI2 (Fig. 4a). Here, the signal acquisition can start immediately after the z-filter^42^ that follows the CP step. Note that signals of protons not coupled to ^19^F can appear in the spectrum even at this point due to the ^1^H–^1^H TOCSY transfer that takes place simultaneously with the heteronuclear CP step.

This pulse sequence can be extended by a dedicated ^1^H–^1^H DIPSI-2 spin-lock propagating the magnetisation transfer to more remote parts of the spin system. Application of two z-filters and ^19^F decoupling ensures that pure inphase ^1^H multiplets are eventually acquired. DIPSI-3,^45^ using 40 μs ^19^F/^1^H pulses, was applied for the CP step covering a ±4 kHz frequency range with >75% efficiency. A slight improvement was achieved with the FLOPSY-16 mixing scheme^46^ covering ±4.7 kHz, *i.e.* 25 ppm of ^19^F resonances on a 400 MHz NMR spectrometer with >65% efficiency relative to the on-resonance signal (Fig. S2†). Further improvements, not explored here, can be achieved by using broadband pulses during the CP step.^47^

An overlay of two 400 MHz 2D ^19^F, ^1^H CP-DIPSI3–DIPSI2 spectra acquired with a 20 ms ^19^F → ^1^H cross-polarisation (red) and an additional 50 ms ^1^H → ^1^H spin-lock (blue) using the pulse sequence of Fig. 4a is presented in Fig. 4b. Both spectra are suitable for the determination of the *J*_HH_ coupling constants. The former spectrum contains pure in phase multiplets of protons H2, 3 and 4 of 1, while the latter spectrum also shows all their other protons. Note the dominance of the H3 signals in the red spectra caused by an effective CP *via* large *J*_H3F3_ (∼50 Hz).

### Fluorine–carbon correlation

Structure determination of sparsely protonated fluorinated molecules, such as heavily substituted aromatic rings, based only on ^1^H and ^19^F chemical shifts and coupling constants could be problematic. Thanks to the far-reaching ^19^F–^13^C couplings (^*n*^*J*_FC_, *n* = 1–5), many ^19^F-coupled ^13^C atoms can be identified by 2D ^19^F, ^13^C correlated experiments such as HMBC or HSQC, making structure determination of such molecules possible. A 2D ^19^F, ^13^C HSQC experiment^33,41^ was not considered in this study mainly because of a larger complexity of the double INEPT transfer. For small molecules, the slower relaxation of single-quantum (HSQC) relative to multiple-quantum (HMBC) coherences does not make a substantial difference to their sensitivity and for mono-fluorinated compounds *F*_1_ singlets are produced by both experiments.

As the ^1^*J*_FC_ coupling constants are large (∼150–250 Hz), while the ^*n*>1^*J*_FC_ typically range from 0 to 50 Hz,^48^ the one-bond (Fig. S3†) and long-range correlation (Fig. 5a) experiments are best performed separately. A single long-range optimised experiment can also yield one-bond correlations if multiple rotations of the ^19^F magnetisation vectors during the evolution interval fall outside of even multiples of 0.5/^1^*J*_FC_. This approach can only be used when values of ^1^*J*_FC_ coupling constants are known, and if dealing with mixtures, their spread is narrow. Values of ^1^*J*_FC_ coupling constants required for such optimisation can be obtained from 1D ^1^H-decoupled ^19^F spectra acquired with a sufficient S/N ratio. Alternatively, accordion optimisation^49^ can be used to obtain simultaneously both types of correlations. Both experiments perform best when ^1^H decoupling is applied during most of the pulse sequence. Such decoupling removes splitting of cross peaks by ^1^H–^13^C couplings in *F*_1_ and by *J*_HF_ in *F*_2_. Resulting *F*_1_ singlets and *F*_2_ anti-phase doublets split by ^19^F–^13^C interactions (Fig. 5b) allow accurate measurement of *J*_FC_ coupling constants that provide valuable structural information.

A comparison of ^19^F chemical shifts of ^13^C isotopomers obtained from 2D ^19^F, ^13^C HMBC spectra with the ^19^F signal in a 1D ^1^H-decoupled ^19^F spectrum yields ^13^C induced ^19^F isotopic shifts (see a large isotopic shift of C3 resonances in Fig. 5b). In aliphatic systems these decrease with the number of bonds separating the two atoms and are generally measurable to up to four bonds separating ^13^C and ^19^F. A careful alignment of the one-bond correlation trace from the pure phase HMBC spectrum and the satellites from the 1D ^19^F spectrum is required to obtain accurate values of these isotopic shifts.

### Proton–carbon–fluorine correlation

^1^H–^1^H and ^1^H–^13^C interactions are the cornerstone of NMR structure determination of small molecules. For fluorinated compounds, the existence of ^1^H–^19^F and ^19^F–^13^C couplings makes this process even more robust. However, for complex mixtures, mapping of these interactions separately, can compromise identification of the nuclei belonging to individual molecules.

This ambiguity can be avoided by correlating all three spin types in a dedicated HCF experiment. There are numerous possibilities for how such an experiment can be designed. Inspired by the 3D HNCA, a pulse sequence for assigning protein backbone resonances,^50^ a ^1^H-detected 3D triple-resonance ^1^H, ^13^C, ^19^F experiment has been proposed previously.^51,52^ This out-and-back 3D experiment contains *J*_FC_ defocusing and refocusing intervals, samples ^19^F and ^13^C chemical shifts indirectly and applies simultaneous ^13^C and ^19^F decoupling during the direct detection of ^1^H. We prefer to use a unidirectional polarisation transfer pathway and direct detection of ^19^F; both of these features are well suited for molecules with a large spread of coupling constants, as is typical for ^19^F–^13^C interactions. The pulse sequence of such an experiment starts with a one-bond ^1^H–^13^C correlation step followed by a ^13^C, ^19^F long-range transfer step. It incorporates a reduced dimensionality approach^53–55^ and samples ^13^C chemical shifts simultaneously with the indirect labelling of ^1^H resonances. The resulting 2D experiment is referred to as (3, 2)D H^1^C^*n*^F, where the superscripts indicate the type of ^13^C and ^19^F interactions (1-one-bond, *n*-long-range) mediating the polarisation transfer (Fig. 6a).

In the (3, 2)D H^1^C^*n*^F experiment, the ^1^H chemical shifts are recorded first, while suppressing the evolution of ^1^H–^1^H and ^1^H–^19^F couplings by a BIRD^r,X^ pulse^56,57^ and a 180° ^19^F pulse applied in the middle of the *t*_1_ period, respectively.

The magnetisation is then transferred in an INEPT step to ^13^C *via* one-bond ^1^H–^13^C couplings, where it is refocused before starting ^1^H decoupling. During the subsequent evolution interval, the ^19^F–^13^C anti-phase magnetisation is developed while the central 180° ^13^C and ^19^F pulses move simultaneously with the *t*_1_ incrementation. This causes modulation of ^1^H chemical shifts by ^13^C offsets, *Ω*_13C_ (=*δ*(^13^C) − ^13^C r.f. carrier frequency) of their directly bonded ^13^C, splitting the signals into doublets centred at the ^1^H chemical shift. The size of ^13^C doublets can be scaled down relative to the *t*_1_ evolution (*κ* factor), keeping the *F*_1_ spectral width small and without any limitations for setting the length of the constant-time ^19^F–^13^C coupling evolution interval, 2*Δ*_5_.

The signal is finally transferred to ^19^F, where it is detected during *t*_2_ under ^1^H decoupling as a pure phase doublet in anti-phase with regard to *J*_FC_ (Fig. 6b).

Interleaved acquisition of two spectra, differing by 90° in the phase of the last 90° ^13^C pulse of the pulse sequence, generates inphase and anti-phase *F*_1_ doublets, respectively, allowing spectra to be simplified by spectral editing^58,59^ as illustrated in Fig. S5.† A pulsed field gradient assisted echo-antiecho protocol is used to obtain pure phase signals in *F*_1_.

Overall, the reduced dimensionality experiment retains the full information content of 3D spectra with substantially increased digital resolution. Due to the use of a single ^*n*^*J*_FC_ evolution interval, sensitivity is also improved relative to the original 3D HCF experiment.^51^ Detecting ^19^F under ^1^H decoupling during *t*_2_ further increases sensitivity of this experiment, while providing values of *J*_FC_ coupling constants. The (3, 2)D H^1^C^*n*^F experiment thus complements the 2D ^19^F, ^13^C HMBC technique discussed above and for protonated carbons correlates unambiguously three atom types, HCF, instead of aiming to achieve the same through a combined interpretation of 2D ^1^H, ^13^C HSQC and 2D ^19^F, ^13^C HMBC spectra, which for complex mixtures, is problematic.

### Structure determination process in ^19^F-centred NMR

**Fig. 7:**
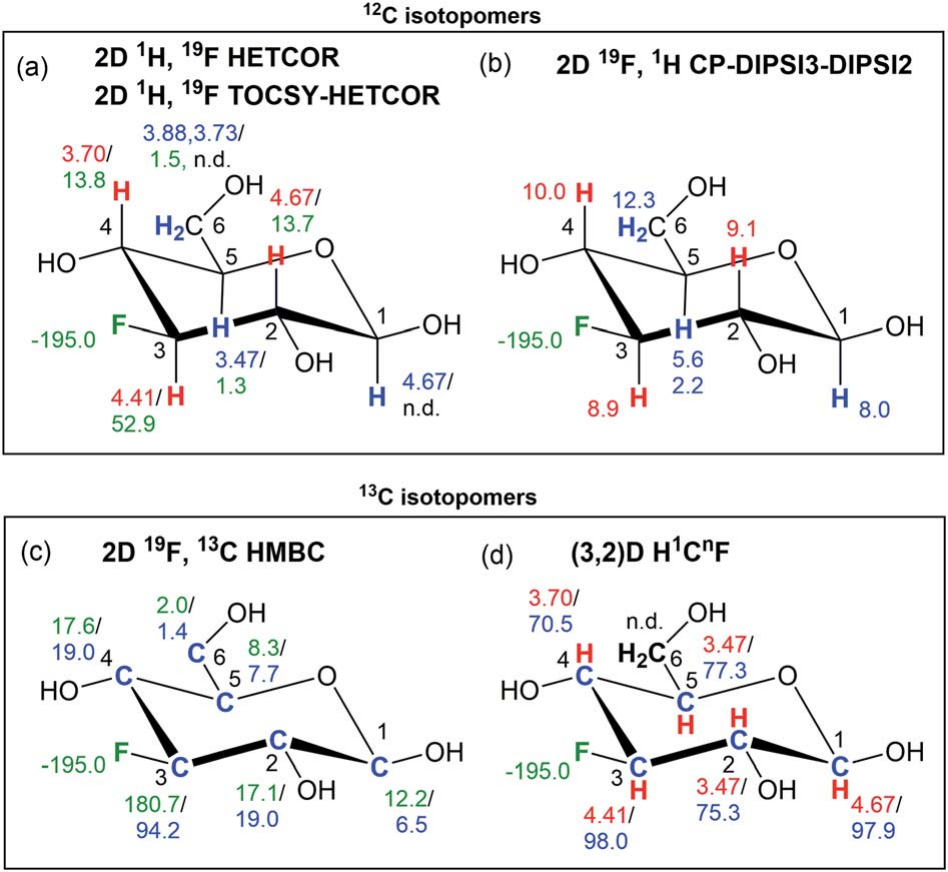
NMR parameters obtained by ^19^F-centred NMR for the β-anomeric form of 1. Chemical shifts, coupling constants and ^13^C isotopic shifts are given in ppm, Hz and ppb, respectively. (a) *δ*_1H_/^*n*^*J*_HF_; red and blue colours indicate correlations obtained from 2D ^1^H, ^19^F HETCOR and 2D ^1^H, ^19^F TOCSY–HETCOR spectra, respectively; (b) *J*_H*i*,H(*i*+1)_, red and blue colour indicates correlation from 2D ^19^F, ^1^H CP-DIPSI3–DIPSI2 without and with the DIPSI-2 extension; (c) ^*n*^*J*_FC_/*Δ*^19^F(^13^C); (d) *δ*_1H_/*δ*_13C_; n.d. – not detected.

This process is briefly summarised with the help of a graphical representation in Fig. 7, using the β-anomeric form of 1 as an example. The ^19^F–^1^H correlations experiments, 2D ^1^H, ^19^F HETCOR and 2D ^1^H, ^19^F TOCSY–HETCOR spectra, together with 1D ^1^H-coupled/decoupled ^19^F spectra provided the parameters summarised in Fig. 7a, while 2D ^19^F, ^1^H CP-DIPSI3–DIPSI2 experiments extended the identified spin system to protons not directly coupled to fluorine (Fig. 7b).

These experiments thus provide ^19^F and ^1^H chemical shift correlations together with ^*n*^*J*_HF_ (*n* = 2–4)^60^ and ^*n*^*J*_HH_ (*n* = 2–3) coupling constants, enabling the start of a structure determination process.

Experiments involving ^19^F–^13^C correlations are very informative. Central to these is the 2D ^19^F, ^13^C HMBC experiment, which provides long-range ^19^F–^13^C correlations and ^*n*^*J*_FC_ coupling constants and in conjunction with a 1D ^1^H decoupled ^19^F spectrum also the ^13^C induced ^19^F isotopic chemical shifts (Fig. 7c). The subsequent (3, 2)D H^1^C^*n*^F experiment provides correlations of HC pairs, in which the carbon is coupled to ^19^F, and if present, a distinction between non-protonated and protonated carbons (Fig. 7d).

Occasionally, a 2D ^1^H, ^19^F HOESY experiment^31,61^ can be used to identify protons not accessible by exploring *J* coupled networks of spins. In general, at this point, the chemical shift assignment and of numerous ^1^H, ^13^C and ^19^F resonances, values of *J*_HF_, *J*_HH_ and *J*_FC_ coupling constants and ^13^C induced ^19^F isotopic shifts are known and the structure determination of fluorine containing moieties can be completed.

For larger molecules, which contain spin systems isolated from those containing ^19^F, the ^19^F-centered approach provides a starting point by identifying protons and carbons that appear in both the ^19^F-centered and the standard ^1^H–^1^H and ^1^H–^13^C 2D chemical shift correlated spectra. These resonances can then be used to extend the structures and connect the fluorinated and non-fluorinated parts of molecules, *e.g. via*^1^H–^1^H NOESY experiments or ^1^H–^13^C HMBC experiments, which can bridge such spin-systems. This approach is particularly beneficial for analyses of mixtures, where the identity of cross peaks belonging to the non-fluorinated parts of the molecule could be difficult to establish.

Although the discussed NMR experiments were developed for mono-fluorinated compounds, they can also be applied to compounds bearing more than one fluorine atom. Nevertheless, the presence of multiple ^19^F atoms should be taken into account when setting up some of the experiments, as the existence of passive ^1^H–^19^F (or ^19^F–^13^C) couplings need to be reflected in the parameters used as outlined in Table S3.†

It should be emphasised, that the ^19^F-centered approach takes full advantage of the high sensitivity of ^19^F to its environment and minute differences in the ^19^F chemical shift of the order of few Hz are sufficient to obtain the kind of information illustrated here on a very simple mixture provided by 1. Application of the ^19^F-centered approach to a very complex mixture of chloramination by-products is presented elsewhere.^30^

## Conclusions

The described methodology is based on a concerted use of several NMR experiments, nevertheless, these can also be used in their own right. Collectively, these experiments represent the most effective NMR approach for the structure determination of mono-fluorinated compounds, particularly those contained in mixtures.

The ^19^F-centred approach developed here is applicable at any point during the lifetime of fluorinated compounds, *e.g.* in analysing reaction mixtures during their production, performing mechanistic studies to understand reaction mechanisms and to optimise chemical reactions, investigating their stability, pharmacokinetics, biodegradation and biotransformation and ultimately to follow their fate in the environment.^62^

## Data availability

The spectra obtained in this study are available here: https://doi.org/10.7488/ds/3422.

## Conflicts of interest

There are no conflicts to declare.

## Supplementary Material

RA-012-D1RA08046F-s001
