# Supplementary material for: 19F-centred NMR analysis of mono-fluorinated compounds
Source: RSC Adv. 2022 Mar 30;12(16):10062–70. doi: 10.1039/d1ra08046f (PMC8966657; doi:10.1039/d1ra08046f)
Supplement: RA-012-D1RA08046F-s001 [file RA-012-D1RA08046F-s001.pdf]

Electronic Supplementary Material (ESI) for RSC Advances.
This journal is © The Royal Society of Chemistry 2022

Supplementary Information

^{19}F-centred NMR analysis of mono-fluorinated compounds

Alan J. R. Smith, Richard York, Dušan Uhrín and Nicholle G. A. Bell*

EaStCHEM School of Chemistry, University of Edinburgh, David Brewster Rd, Edinburgh, EH9 3FJ, UK.

Corresponding Author:
* Nicholle Bell - School of Chemistry, University of Edinburgh, Joseph Black Building, Kings Buildings, David Brewster Road, EH93FJ. Email: Nicholle.Bell@ed.ac.uk, Orcid: 0000-0001-7887-2659

Table of Contents

Table S1. Parameters of NMR experiments performed on the 3-deoxy-3-fluoro-D-glucose, **1**. 2

Table S2. NMR parameters of 3-deoxy-3-fluoro-d-glucose, **1**. 3

Table S3. Setting up the ^{19}F-centred NMR experiments on compounds bearing more than one fluorine atom. 4

Figures

Figure S1. Inversion profile of a 1 ms 180° CHIRP pulse....................5

Figure S2. Transfer profiles of the DIPSI3 and FLOPSY-16 cross polarisation....................5

Figure S3. Pulse sequence of a ^{19}F-detected 2D ^{19}F, ^{13}C HMBC experiment optimised for $^{1}J_{FC}$ correlations 6

Figure S4. A 2D ^{19}F-detected 2D ^{19}F, ^{13}C HMBC spectrum optimised for $^{1}J_{FC}$ correlations…6

Figure S5. Editing of (3, 2)D H^{1}C^{n}F spectra of **1**....................7

Bruker pulse programs

Pulse program PP.1. Variable-time z-filtered ^{19}F-detected 2D ^{1}H, ^{19}F HETCOR 8

Pulse program PP.2. Variable-time ^{19}F-detected 2D ^{1}H, ^{19}F TOCSY-HETCOR....................11

Pulse program PP.3. 2D ^{19}F, ^{1}H CP-DIPSI3-DIPSI2....................15

Pulse program PP.4. 2D ^{19}F, ^{13}C(^{15}N) HMBC optimised for $^{n}J_{FC}$ ($^{n}J_{FN}$) coupling constants.21

Pulse program PP.5. 2D ^{19}F, ^{13}C HMBC optimised for $^{1}J_{FC}$ coupling constants....................24

Pulse program PP.6. (3, 2)D H^{1}C^{n}F 27

Table S1. Parameters of NMR experiments performed on the 3-deoxy-3-fluoro-D-glucose, **1.**

Parameter [a] /Experiment	*J* evolution delay /ms	SW_1 | SW_2 /ppm	TD_1 | TD_2 /points	AQ_1 | AQ_2 /ms | /s	Overall time /h
2D ^{19}F-detected VT-^{1}H,^{19}F HETCOR	$2\Delta_2 = 25$	2.5 | 8	128 | 4096	64 | 0.68	0.33
2D ^{19}F-detected VT, z-filtered ^{1}H,^{19}F HETCOR	$2\Delta_2 = 25$	2.5 | 8	128 | 4096	64 | 0.68	0.33
2D ^{19}F-detected ^{1}H,^{19}F TOCSY-HETCOR	$2\Delta_2 = 25$	2.5 | 8	128 | 4096	64 | 0.68	0.33
2D ^{19}F, ^{1}H CP-DIPSI3-DIPSI2	20 (F→H) 50 (H→H)	8 | 3	128 | 2048	21 | 0.853	0.5
2D ^{19}F,^{13}C HMBC ($^{n}J_{FC}$)	2d6 = 25	60 | 7	256 | 2048	21 | 0.388	0.5
2D ^{19}F,^{13}C HMBC ($^{1}J_{FC}$)	2d6 = 2.8	60 | 7	256 | 2048	21 | 0.388	0.5
[b](3, 2)D $H^{1}C^{n}F$	$2\Delta_5 = 27.8$	4.2 | 7.0	320 | 2048	0.097 | 0.38	1.74

[a] Relaxation time: 1.6s; number of scans per increment: 4. [b]parameters of the overall interleaved experiment.

Table S2. NMR parameters of 3-deoxy-3-fluoro-d-glucose, **1**.

	$\delta(^{1}H)$ /ppm[a]	$\delta(^{19}F)$ /ppm[b]	$\delta(^{13}C)$ /ppm[b]	J_{HxHy} /Hz[a] (x, y)	J_{HF} /Hz[b]	J_{FC} /Hz[b]	$\Delta^{19}F(^{13}C)$[b,c] /ppb	$\delta(^{1}H)$ /ppm[a]	$\delta(^{19}F)$ /ppm[b]	$\delta(^{13}C)$ /ppm[b]	J_{HxHy} /Hz[a] (x, y)	J_{HF} /Hz[b]	J_{FC} /Hz[b]	$\Delta^{19}F(^{13}C)$[b,c] /ppb
Atom	α-anomer							β-anomer						
1	5.25	-	94.9	3.9 (1,2)	3.7	10.7	7.1	4.67	-	97.9	8.0 (1,2)	n.d.	12.2	6.5
2	3.79	-	72.7	3.9 (2,1)<br>9.4 (2,3)	13.1	16.7	18.9	3.51	-	75.3	8.0 (2,1)<br>9.1 (2,3)	13.7	17.1	19.0
3	4.59	-199.9	97.7	9.5 (3,2)<br>8.9 (3,4)	54.3	178.7	94.6	4.41	-195.0	98.0	9.1(3,2)<br>8.8 (3,4)	52.9	180.7	94.2
4	3.71	-	70.6	8.9 (4,3)<br>10.2(4,5)	13.8	17.8	19.7	3.70	-	70.5	8.9 (4,3)<br>10.0(4,5)	13.8	17.6	19.0
5	3.84	-	73.5	10.2(5,4)<br>2.3 (5,6)<br>4.9 (5,6’)	n.d.	7.3	6.7	3.47	-	77.3	10.0(5,4)<br>2.2 (5,6)<br>5.6 (5,6’)	1.3	8.3	7.7
6	3.83	-	63.1	2.3 (6,5)<br>12.2(6,6’)	1.8	1.5	1.3	3.88	-	62.9	2.2 (6,5)<br>12.4(6,6’)	1.5	2.0	1.4
6’	3.77	-	-	5.0 (6’,5)<br>12.3(6’,6)	n.d.	-	-	3.73	-	-	5.5 (6’,5)<br>12.4(6’,6)	n.d.	-	-

[a]800 MHz data; [b] 400 MHz data; [c] $\Delta^{19}F(^{13}C) = 10^{3} \times [\nu_{\delta}(^{19}F_{12C}) - \nu_{\delta}(^{19}F_{13C})]/ \nu_{L}(^{19}F)$ where ν_{δ} and ν_{L} are given in Hz and MHz, respectively; $\nu_{L}(^{19}F)$ = 376.4984 MHz.

The $^{n}J_{FH}$, $^{n}J_{HH}$ and $^{n}J_{FC}$ coupling constants listed in Table S2 are determined with a precision of ± 0.1 Hz due to sufficiently long acquisition times (> 0.4 s) in the directly detected dimension of 2D experiments. In case of the $^{n}J_{FH}$ in the z-filtered 2D ^{1}H, ^{19}F HETCOR spectra, achieving such precision requires to work with 1D ^{1}H coupled ^{19}F spectra, as the partial cancellation of antiphase lines in ^{19}F multiplets of 2D spectra can affect the accuracy of the determined coupling constants.

Table S3. Setting up the ^{19}F-centred NMR experiments on compounds bearing more than one fluorine atom.

<table>
<tr><td>Experiment</td><td>z-filtered 2D ^{1}H, ^{19}F HETCOR</td><td>2D ^{1}H, ^{19}F TOCSY-HETCOR</td><td>2D ^{19}F, ^{1}H CP-DIPSI3-DIPSI2</td><td>2D ^{19}F, ^{13}C HMBC</td><td>(3,2)D H^{1}C^{n}F correlation</td></tr>
<tr><td>^{19}F resonances are mutually coupled</td><td colspan="2">Issue: passive $^{n}J_{FH}$ are evolving during 2Δ_2.
Solution: may need to shorten Δ_2 to optimise sensitivity.
Difference: inphase splitting due to $^{n}J_{FF}$ couplings in F_2.</td><td>$F_i \rightarrow F_j$ transfer can occur; a cross over between the F_i and F_j spin systems is possible.</td><td>Mixed phase, ($^{n}J_{FF}$) multiplets in F_2</td><td>Issue: passive $^{n}J_{FC}$ are evolving during 2Δ_5.
Solution: shorten Δ_5 to optimise sensitivity.
Difference: inphase splitting due to $^{n}J_{FF}$ couplings in F_2.</td></tr>
<tr><td>^{19}F resonances only couple to ^{1}H and ^{13}C atoms</td><td colspan="2">Issue: passive $^{n}J_{FH}$ are evolving during 2Δ_2.
Solution: may need to shorten Δ_2 to optimise sensitivity.</td><td>N.D.</td><td>N.D.</td><td>Issue: passive $^{n}J_{FC}$ are evolving during 2Δ_5.
Solution: shorten Δ_5 to optimise sensitivity.</td></tr>
</table>

N.D. no difference from molecules with one ^{19}F atom.

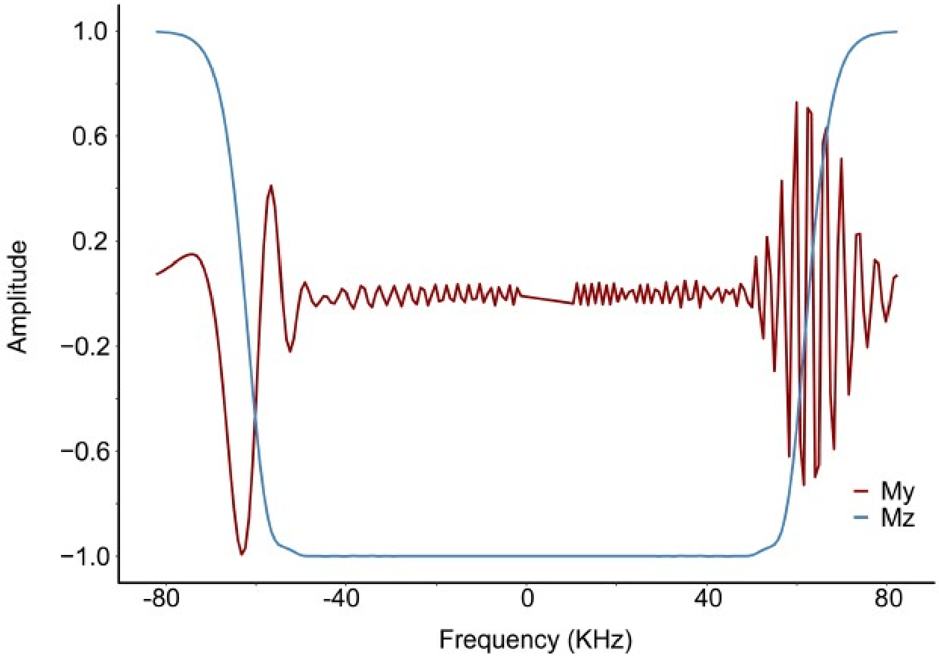

Fig. S1. Inversion profile of a 1 ms 180° CHIRP pulse[1] (140 kHz sweep, 20% smoothing) applied with a peak power of 10.3 kHz. This pulse inverts perfectly a 100 kHz spectral ^{19}F window (266 ppm for a 400 MHz spectrometer).

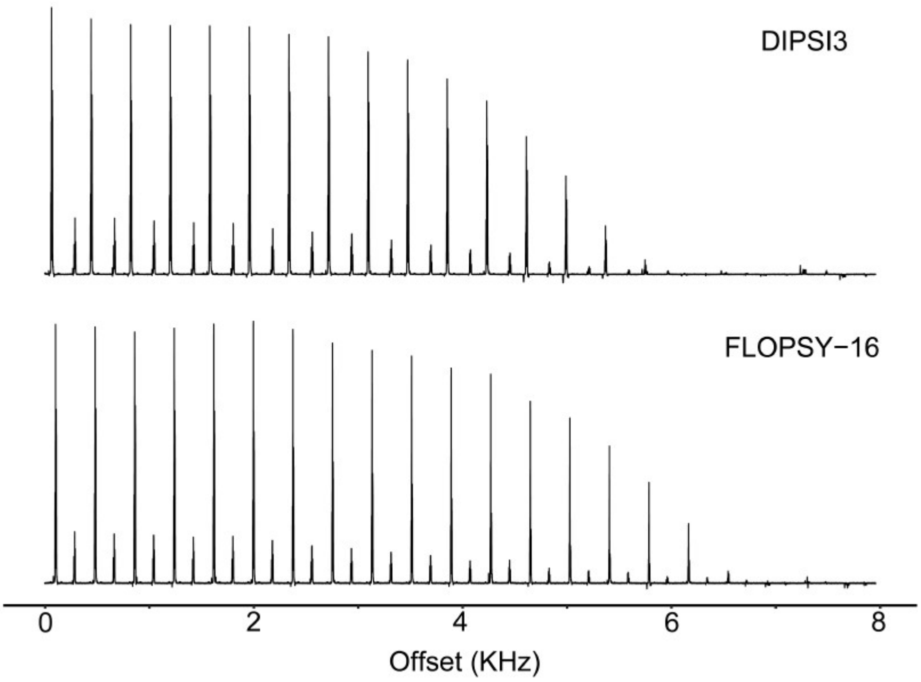

Fig. S2. Excitation profiles for DIPSI-3 and FLOPSY-16 CP spin locks produced using 40 μs matched ^1H and ^{19}F pulses.

[1] J. M. Bohlen and G. Bodenhausen, *Journal of Magnetic Resonance Series A*, 1993, **102**, 293-301.

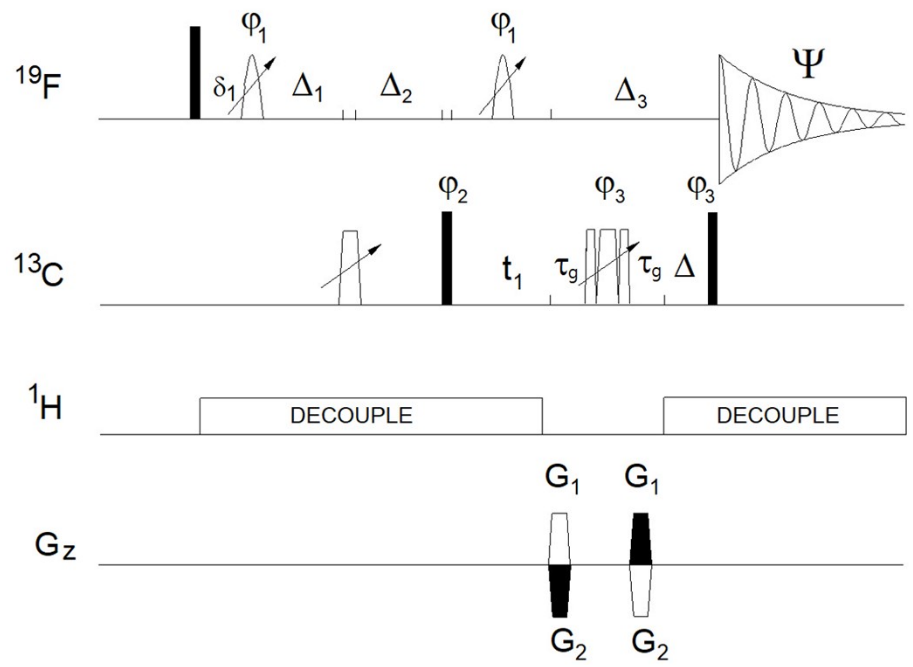

Figure S3. Pulse sequence of ^{19}F-detected 2D ^{19}F, ^{13}C HMBC experiment optimised for $^{1}J_{FC}$ correlations. The thin filled rectangles represent high power 90° ^{19}F (p1) or ^{13}C (p3) pulses. The 1 ms adiabatic CHIRP pulses (p44) applied to ^{19}F are indicated by an inclined arrow. A 500 μs CHIRP pulse (p14) and 2 ms composite CHIRP pulse (p24) were applied to ^{13}C. Unless stated otherwise, the r.f. pulses were applied from the *x*-axis. The delays were as follows: d6 = $0.5/^{1}J_{FC}$; Δ = p44; δ_1 = 20μs; Δ_3 =2*p16+2*d16+p24+Δ+8μs; $\Delta_1 = (\Delta_3 - \text{p14} - \text{d6})/2 + (2/\pi)\text{*p1} + \delta_1$; $\Delta_2 = (\Delta_3 - \text{p14} + \text{d6})/2$; $t_1(0)$ is the initial t_1 evolution delay time = 0.5*in0, where in0 is the t_1 increment. G_1 = 80%; G_2 = cnst30*G_1, where cnst30 = (1-sfo2/sfo1)/(1+sfo2/sfo1) and sfo1 and sfo2 are ^{19}F and ^{13}C frequencies, respectively. φ_1 = 2x, 2(-x); φ_2 = x, -x; φ_3 = 4x, 4(-x); Ψ = 2(x, -x), 2(-x, x). Echo-anti echo protocol was used with PFGs changing the sign between real and imaginary increments. Phases φ_2 and Ψ were incremented by 180° together with the sign

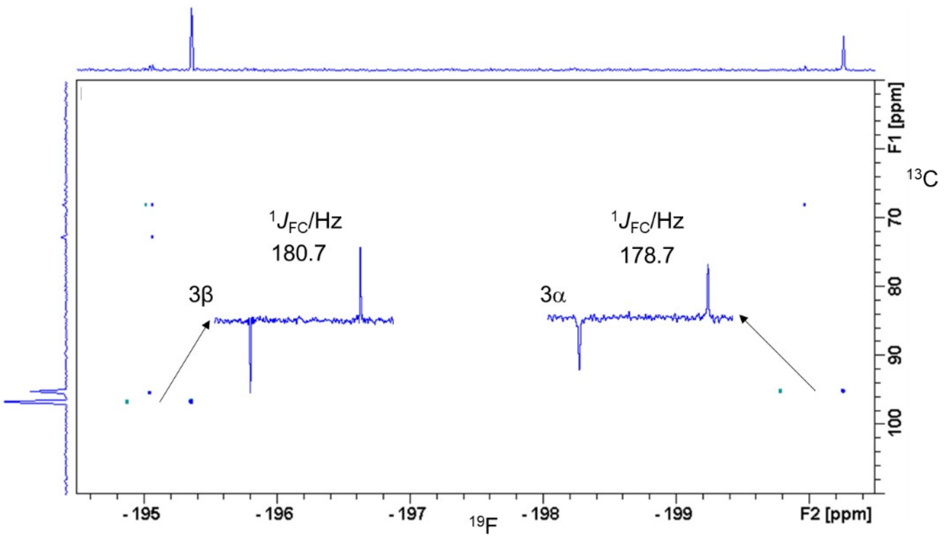

change.

Figure S4. A 2D ^{19}F-detected 2D ^{19}F, ^{13}C HMBC spectrum optimised for $^{1}J_{FC}$ correlations acquired using the pulse sequence of Fig. S3. Anti-phase F_2 doublets through C3 of α/β **1** show $^{1}J_{FC}$ coupling constants.

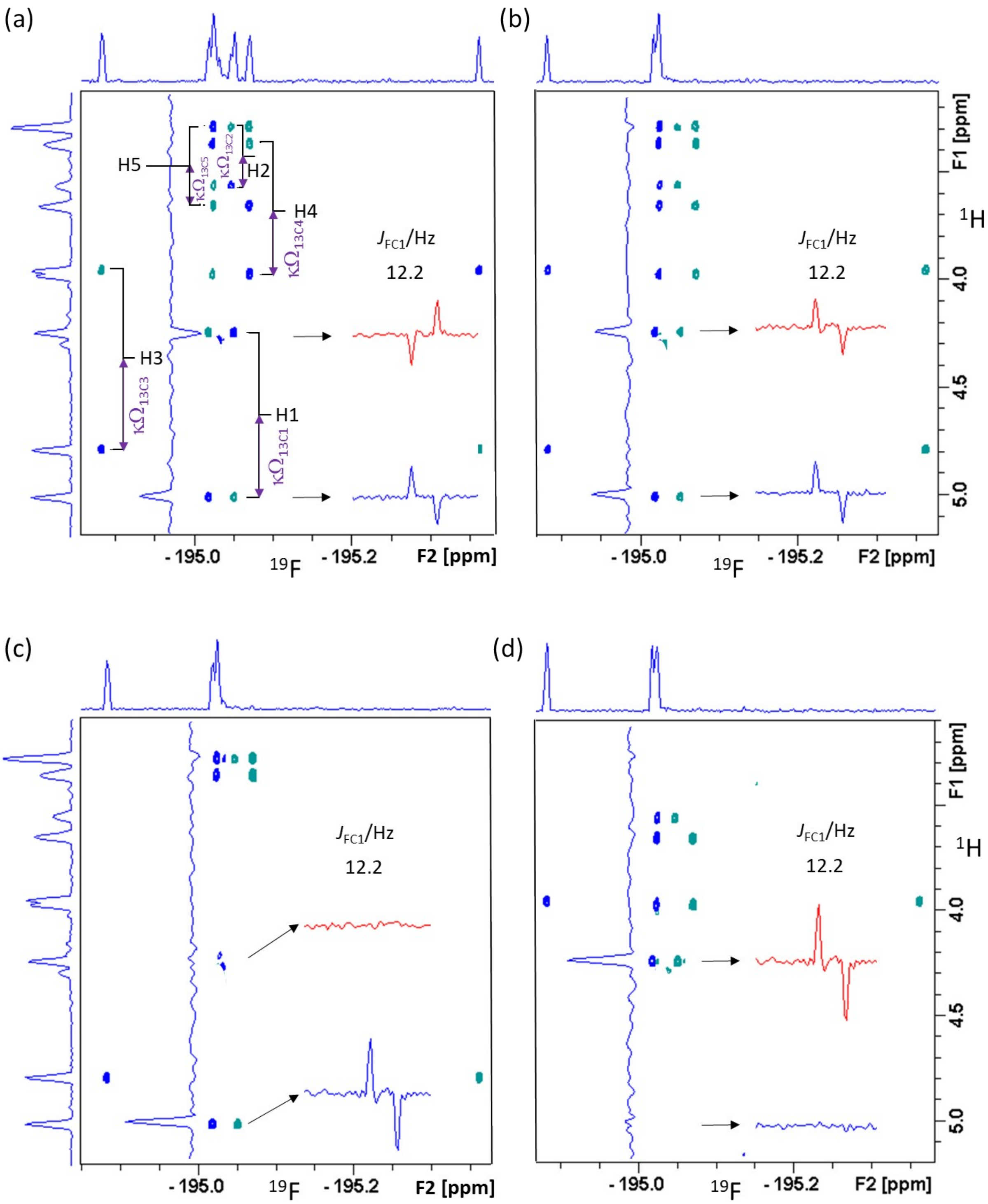

Figure S5. Editing of (3, 2)D H^{1}C^{n}F spectra of **1** acquired using the pulse sequence of **Fig. 6** showing the cross peaks of the β-anomer with (a) anti-phase and (b) in phase F_1 splittings. In all spectra, the insets contain vertical and horizontal traces through the H1, F cross peaks. The ^{1}H chemical shift of protons directly attached to ^{13}C atoms and the associated $\kappa\Omega_{13C}$ frequencies are indicated. Antiphase doublets in F_2 show J_{FC} coupling constants. (c) and (d)

spectra represent the sum and the difference of the (a) and (b) spectra, respectively. Horizontal and vertical internal projections are shown on the top and at the side of all spectra, respectively.

Pulse program PP.1. Variable-time z-filtered ^{19}F-detected 2D ^{1}H, ^{19}F HETCOR

```
;1H19F_VT_HETCOR.2.as
;19F-detected variable time z-filtered 2D 1H,19F chemical shift
correlation
;phase sensitive, pure phase multiplets in F1
;
;A.J.R. Smith, R. York, D. Uhrín and N.G.A. Bell (to be completed)
;M.R. Bendall & D.T. Pegg, J. Magn. Reson. 53, 144 (1983)
;T.T. Nakashima et al., J. Magn. Reson. 59, 124 (1984)
;M.J. Thrippleton & J. Keeler, Angew. Chem. Int. Ed. 42, 3938-3941
(2003)
;
;$CLASS=HighRes
;$DIM=2D
;$TYPE=
;$SUBTYPE=
;$COMMENT=


#include <Avance.incl>
#include <Delay.incl>
#include <Grad.incl>

"p4=p3*2"
"d11=30m"
"d12=20u"
"d13=3u"
"DELTA2=1/4*cnst10";in the presence of JHH set substantially shorter

"in0=inf1/2"

# ifdef F1180
"d0=0.5*in0"
"DELTA1=p44"
# else
"d0=3u"
"DELTA1=(2*d0+p44)"
# endif

"acqt0=-p1*2/3.1416"

1 ze
  d12 pl1:f1 pl2:f2
2 d11

  d12 BLKGRAD
  d1 pl1:f1
  50u UNBLKGRAD
  p16:gp1
  d16
  p1 ph4
```

```
  2u
  p1 ph5
  4u
  p16:gp2
  d16

  (p3 ph1):f2   ; this is the real start
  d0 pl8:f1
  (p44:sp30 ph4):f1
  d0
  (p4 ph2):f2
  DELTA1

  (p3 ph6):f2
  10u gron0
  d12 pl0:f2
  (p32:sp29 ph4):f2
  20u groff
  d16
  p16:gp4
  d16 pl2:f2
  (p3 ph4):f2

  DELTA2
  (center (p44:sp30 ph4):f1 (p4 ph4):f2)
  DELTA2 pl1:f1

  (p3 ph3):f2
  p16:gp3
  d16
  (p1 ph4):f1
  go=2 ph31
  d11 mc #0 to 2 F1PH(calph(ph1, +90), caldel(d0, +in0))
  d12 BLKGRAD
exit

ph1=0 2
ph2=0 0 0 0 2 2 2 2
ph3=1 1 3 3
ph4=0
ph5=1
ph6=2
ph31=0 2 2 0


;pl1 : f1 channel (19F) - power level for pulse (default)
;pl2 : f2 channel (1H)- power level for pulse (default)
;pl0: f1 channel - no power on f1 (1000 db)
;p1: f1 channel (19F)- 90 degree high power pulse
;p3 : f2 channel (1H) -  90 degree high power pulse
;p4 : f2 channel (1H) (180 degree high power pulse
;p44 : 180 deg BB 19F pulse
;spnam30: Crp140,1,20.1
;spdB30: power level for 180 degree 19F shape pulse
;p44 ; broad band 180 degree 19F shape pulse
;p32: f1 channel - 180 degree shaped pulse (adiabatic)    [20 msec]
```

```
;     smoothed chirp (sweepwidth, 20% smoothing, 10000 points)
;sp29: f1 channel - shaped pulse (adiabatic)
;spnam29:
;d0 : incremented delay (2D)                              [5 usec]
;d1 : relaxation delay; 1-5 * T1
;cnst10 : JHF coupling constant
;DELTA1 = 1/4*cnst10
;d11: delay for disk I/O                                  [30 msec]
;d12: delay for power switching                           [20 usec]
;inf1: 1/SW(H) = 2 * DW(H)
;in0: 1/(2 * SW(H)) = DW(H)
;nd0: 2
;ns: 2 * n
;ds: 4
;td1: number of experiments
;FnMODE: States-TPPI, TPPI, States or QSEQ

;Processing

;if zgoptns -DF1180
;PHC0(F1): 90
;PHC1(F1): -180
;FCOR(F1): 1

;else
;PHC0(F1): 0
;PHC1(F1): 0
;FCOR(F1): 0.5
```

Pulse program PP.2. Variable-time ^{19}F-detected 2D ^{1}H, ^{19}F TOCSY-HETCOR

```
;1H19F_TOCSY_HETCOR.as
;19F-detected variable time 2D 1H,19F chemical shift correlation
;with a preceding HH TOCSY step

;A.J.R. Smith, R. York, D. Uhrín and N.G.A. Bell (to be completed)
;M.R. Bendall & D.T. Pegg, J. Magn. Reson. 53, 144 (1983)
;T.T. Nakashima et al., J. Magn. Reson. 59, 124 (1984)
;M.J. Thrippleton & J. Keeler, Angew. Chem. Int. Ed. 42, 3938-3941
(2003)
;
;$CLASS=HighRes
;$DIM=2D
;$TYPE=
;$SUBTYPE=
;$COMMENT=

#include <Avance.incl>
#include <Delay.incl>
#include <Grad.incl>

"p4=p3*2"
"d11=30m"
"d12=20u"
"DELTA2=1/4*cnst10"

"in0=inf1/2"

# ifdef F1180
"d0=0.5*in0"
"DELTA1=p44"
# else
"d0=3u"
"DELTA1=2*d0+p44"
# endif

"FACTOR1=(d9/(p6*115.112))/2"
"l1=FACTOR1*2"

"acqt0=-p1*2/3.1416"

1 ze
2 d11
  d1 pl2:f2 pl1:f1 BLKGRAD

  (p3 ph1):f2
  d0 pl8:f1
  (p44:sp30 ph4):f1
  d0
  (p4 ph4):f2
  DELTA1 UNBLKGRAD
  (p3 ph2):f2
  p16:gp1
  d16 pl10:f2
```

```
    ;begin DIPSI2
4  (p6*3.556 ph23):f2
   (p6*4.556 ph25):f2
   (p6*3.222 ph23):f2
   (p6*3.167 ph25):f2
   (p6*0.333 ph23):f2
   (p6*2.722 ph25):f2
   (p6*4.167 ph23):f2
   (p6*2.944 ph25):f2
   (p6*4.111 ph23):f2

   (p6*3.556 ph25):f2
   (p6*4.556 ph23):f2
   (p6*3.222 ph25):f2
   (p6*3.167 ph23):f2
   (p6*0.333 ph25):f2
   (p6*2.722 ph23):f2
   (p6*4.167 ph25):f2
   (p6*2.944 ph23):f2
   (p6*4.111 ph25):f2

   (p6*3.556 ph25):f2
   (p6*4.556 ph23):f2
   (p6*3.222 ph25):f2
   (p6*3.167 ph23):f2
   (p6*0.333 ph25):f2
   (p6*2.722 ph23):f2
   (p6*4.167 ph25):f2
   (p6*2.944 ph23):f2
   (p6*4.111 ph25):f2

   (p6*3.556 ph23):f2
   (p6*4.556 ph25):f2
   (p6*3.222 ph23):f2
   (p6*3.167 ph25):f2
   (p6*0.333 ph23):f2
   (p6*2.722 ph25):f2
   (p6*4.167 ph23):f2
   (p6*2.944 ph25):f2
   (p6*4.111 ph23):f2
   lo to 4 times l1
    ;end DIPSI2

   10u gron0 pl0:f2 pl1:f1
   (p32:sp29 ph4):f2
   20u groff
   d16
   p1 ph4
   2u
   p1 ph5
   p16:gp2
   d16 pl2:f2 pl8:f1

   (p3 ph4):f2 ;spin-echo to develop antiphase HF magnetization
   DELTA2
   (center (p44:sp30 ph4) (p4 ph4):f2 )
```

```
  DELTA2 pl1:f1
  (p3 ph3):f2         ;transfer of polarisation
  p16:gp3
  d16
  p1 ph4
  go=2 ph31
  d11 mc #0 to 2 F1PH(calph(ph1, +90), caldel(d0, +in0))
  d12 BLKGRAD
exit


ph1=0 2
ph2=0 0 0 0 2 2 2 2
ph3=1 1 3 3
ph4=0
ph5=1
ph23=3
ph25=1
ph31=0 2 2 0 2 0 0 2


;pl1 : f1 channel (19F) - power level for pulse (default)
;pl2 : f2 channel (1H)- power level for pulse (default)
;pl0: f1 channel - no power on f1 (1000 db)
;p1: f1 channel (19F)- 90 degree high power pulse
;p3 : f2 channel (1H) -  90 degree high power pulse
;p4 : f2 channel (1H) (180 degree high power pulse
;pl8: f2 channel - no power on f1 (1000 db)
;p44 : 180 deg BB 19F pulse
;spnam30: Crp140,1,20.1
;spdB30: power level for 180 degree 19F shape pulse
;p44 ; broad band 180 degree 19F shape pulse
;p6 : f2 channel - 90 degree TOCSY pulse
;p16 : homospoil/gradient pulse (1msec)
;d0 : incremented delay (2D)                         [3 usec]
;d1 : relaxation delay; 1-5 * T1
;d11: delay for disk I/O                             [30 msec]
;d12: delay for power switching                      [20 usec]
;DELTA3=1/4*cnst10
;cnst10 : JHF coupling constant
;DELTA2 = 1/4*cnst10
;d9 : tocsy mixing time
;inf1: 1/SW(H) = 2 * DW(H)
;in0: 1/(2 * SW(H)) = DW(H)
;nd0: 2
;ns: 2 * n
;ds: 4
;td1: number of experiments
;FnMODE: States-TPPI, TPPI, States or QSEQ

;Processing

;if zgoptns -DF1180
;PHC0(F1): 90
;PHC1(F1): -180
;FCOR(F1): 1
```

```
;else
;PHC0(F1): 0
;PHC1(F1): 0
;FCOR(F1): 0.5
```

Pulse program PP.2. 2D ^{19}F, ^{1}H CP-DIPSI3-DIPSI2

```
;19F1H_CP.as

;2D 1H-detected FH heteronuclear chemical shift correlation;
;phase sensitive using 19F-1H cross polarisation and an optional
;HH DIPSI2 transfer

;A.J.R. Smith, R. York, D. Uhrín and N.G.A. Bell(to be completed)
;Hu, H.;  Kulanthaivel, P.; Krishnamurthy, K.,
;J.Org.Chem. 2007, 72 (16), 6259-6262.

;$CLASS=HighRes
;$DIM=2D
;$TYPE=
;$SUBTYPE=
;$COMMENT=


#include <Avance.incl>
#include <Delay.incl>
#include <Grad.incl>

"p2=2*p1"
"p4=p3*2"
"d11=30m"
"d12=20u"
"d13=d12+p3*2/3.1416"

"in0=inf1/2"

# ifdef F1180
"d0=0.5*in0"
"DELTA=p2"
# else
"d0=3u"
"DELTA=2*d0+p2"
# endif

"FACTOR1=(d9/(p25*217.3))" ;d9 CP time
"l1=FACTOR1"

"FACTOR2=(d10/(p6*115.112))/2"   ;d10 HH spinlock time
"l2=FACTOR2*2"

"acqt0=-p1*2/3.1416"


1 ze
2 d11
  d12 do:f2

# ifdef PRESAT
  d12 BLKGRAD pl9:f1
  d1 cw:f1 ph29
  4u do:f1
```

```
# else
  d1
# endif

  10u pl1:f1
  d12 UNBLKGRAD

  p16:gp1
  d16 pl2:f2
  p1 ph13
  2u
  p1 ph25
  p16:gp2
  d16

  (p3 ph1):f2    ;this is the real start
  d12 pl8:f2
  (p44:sp30 ph2):f2
  d13
  d0
  (p2 ph4):f1
  d0
  (p44:sp30 ph2):f2
  DELTA pl10:f1 pl11:f2

  (p8 ph11):f2

                ;begin DIPSI3
4 (p25*2.722 ph11):f1 (p25*2.722 ph13):f2
  (p25*4.389 ph12):f1 (p25*4.389 ph14):f2
  (p25*2.778 ph11):f1 (p25*2.778 ph13):f2
  (p25*3.056 ph12):f1 (p25*3.056 ph14):f2
  (p25*0.333 ph11):f1 (p25*0.333 ph13):f2
  (p25*2.556 ph12):f1 (p25*2.556 ph14):f2
  (p25*4.000 ph11):f1 (p25*4.000 ph13):f2
  (p25*2.722 ph12):f1 (p25*2.722 ph14):f2
  (p25*4.111 ph11):f1 (p25*4.111 ph13):f2
  (p25*3.778 ph12):f1 (p25*3.778 ph14):f2
  (p25*3.889 ph11):f1 (p25*3.889 ph13):f2
  (p25*2.889 ph12):f1 (p25*2.889 ph14):f2
  (p25*3.000 ph11):f1 (p25*3.000 ph13):f2
  (p25*0.333 ph12):f1 (p25*0.333 ph14):f2
  (p25*2.500 ph11):f1 (p25*2.500 ph13):f2
  (p25*4.050 ph12):f1 (p25*4.050 ph14):f2
  (p25*2.830 ph11):f1 (p25*2.830 ph13):f2
  (p25*4.389 ph12):f1 (p25*4.389 ph14):f2


  (p25*2.722 ph12):f1 (p25*2.722 ph14):f2
  (p25*4.389 ph11):f1 (p25*4.389 ph13):f2
  (p25*2.778 ph12):f1 (p25*2.778 ph14):f2
  (p25*3.056 ph11):f1 (p25*3.056 ph13):f2
  (p25*0.333 ph12):f1 (p25*0.333 ph14):f2
  (p25*2.556 ph11):f1 (p25*2.556 ph13):f2
  (p25*4.000 ph12):f1 (p25*4.000 ph14):f2
  (p25*2.722 ph11):f1 (p25*2.722 ph13):f2
```

```
  (p25*4.111 ph12):f1 (p25*4.111 ph14):f2
  (p25*3.778 ph11):f1 (p25*3.778 ph13):f2
  (p25*3.889 ph12):f1 (p25*3.889 ph14):f2
  (p25*2.889 ph11):f1 (p25*2.889 ph13):f2
  (p25*3.000 ph12):f1 (p25*3.000 ph14):f2
  (p25*0.333 ph11):f1 (p25*0.333 ph13):f2
  (p25*2.500 ph12):f1 (p25*2.500 ph14):f2
  (p25*4.050 ph11):f1 (p25*4.050 ph13):f2
  (p25*2.830 ph12):f1 (p25*2.830 ph14):f2
  (p25*4.389 ph11):f1 (p25*4.389 ph13):f2

  (p25*2.722 ph12):f1 (p25*2.722 ph14):f2
  (p25*4.389 ph11):f1 (p25*4.389 ph13):f2
  (p25*2.778 ph12):f1 (p25*2.778 ph14):f2
  (p25*3.056 ph11):f1 (p25*3.056 ph13):f2
  (p25*0.333 ph12):f1 (p25*0.333 ph14):f2
  (p25*2.556 ph11):f1 (p25*2.556 ph13):f2
  (p25*4.000 ph12):f1 (p25*4.000 ph14):f2
  (p25*2.722 ph11):f1 (p25*2.722 ph13):f2
  (p25*4.111 ph12):f1 (p25*4.111 ph14):f2
  (p25*3.778 ph11):f1 (p25*3.778 ph13):f2
  (p25*3.889 ph12):f1 (p25*3.889 ph14):f2
  (p25*2.889 ph11):f1 (p25*2.889 ph13):f2
  (p25*3.000 ph12):f1 (p25*3.000 ph14):f2
  (p25*0.333 ph11):f1 (p25*0.333 ph13):f2
  (p25*2.500 ph12):f1 (p25*2.500 ph14):f2
  (p25*4.050 ph11):f1 (p25*4.050 ph13):f2
  (p25*2.830 ph12):f1 (p25*2.830 ph14):f2
  (p25*4.389 ph11):f1 (p25*4.389 ph13):f2

  (p25*2.722 ph11):f1 (p25*2.722 ph13):f2
  (p25*4.389 ph12):f1 (p25*4.389 ph14):f2
  (p25*2.778 ph11):f1 (p25*2.778 ph13):f2
  (p25*3.056 ph12):f1 (p25*3.056 ph14):f2
  (p25*0.333 ph11):f1 (p25*0.333 ph13):f2
  (p25*2.556 ph12):f1 (p25*2.556 ph14):f2
  (p25*4.000 ph11):f1 (p25*4.000 ph13):f2
  (p25*2.722 ph12):f1 (p25*2.722 ph14):f2
  (p25*4.111 ph11):f1 (p25*4.111 ph13):f2
  (p25*3.778 ph12):f1 (p25*3.778 ph14):f2
  (p25*3.889 ph11):f1 (p25*3.889 ph13):f2
  (p25*2.889 ph12):f1 (p25*2.889 ph14):f2
  (p25*3.000 ph11):f1 (p25*3.000 ph13):f2
  (p25*0.333 ph12):f1 (p25*0.333 ph14):f2
  (p25*2.500 ph11):f1 (p25*2.500 ph13):f2
  (p25*4.050 ph12):f1 (p25*4.050 ph14):f2
  (p25*2.830 ph11):f1 (p25*2.830 ph13):f2
  (p25*4.389 ph12):f1 (p25*4.389 ph14):f2

  lo to 4 times l1
  ;end of DIPSI3
  (p8 ph4):f1

  p25 ph3

# ifdef DIPSI2
```

```
  10u gron0
  (p32:sp29 ph4):f1
  20u groff
  d16 pl10:f1

      ;begin DIPSI2
5 p6*3.556 ph23
  p6*4.556 ph25
  p6*3.222 ph23
  p6*3.167 ph25
  p6*0.333 ph23
  p6*2.722 ph25
  p6*4.167 ph23
  p6*2.944 ph25
  p6*4.111 ph23

  p6*3.556 ph25
  p6*4.556 ph23
  p6*3.222 ph25
  p6*3.167 ph23
  p6*0.333 ph25
  p6*2.722 ph23
  p6*4.167 ph25
  p6*2.944 ph23
  p6*4.111 ph25

  p6*3.556 ph25
  p6*4.556 ph23
  p6*3.222 ph25
  p6*3.167 ph23
  p6*0.333 ph25
  p6*2.722 ph23
  p6*4.167 ph25
  p6*2.944 ph23
  p6*4.111 ph25

  p6*3.556 ph23
  p6*4.556 ph25
  p6*3.222 ph23
  p6*3.167 ph25
  p6*0.333 ph23
  p6*2.722 ph25
  p6*4.167 ph23
  p6*2.944 ph25
  p6*4.111 ph23
  lo to 5 times l2
      ;end DIPSI2
# endif

  10u gron0*1.333
  (p32*0.75:sp29 ph4):f1
  20u groff
  d16 pl1:f1 pl16:f2
  p16:gp3
  d16
```

```
  p1 ph6

  go=2 ph31 cpd3:f2
  d11 do:f2 mc #0 to 2 F1PH(calph(ph1, -90), caldel(d0, +in0))

  4u BLKGRAD
exit

ph1=1 3
ph2=0 0 0 0 2 2 2 2
ph3=1 1 3 3
ph4=0
ph5=0
ph6=2
ph11=0
ph12=2
ph13=0
ph14=2
ph23=3
ph25=1
ph29=0
ph31=0 2 2 0

;pl1 : f1 channel - power level for pulse (default)
;pl2 : f2 channel - power level for pulse (default)
;pl10 : f1 channel - power level for DIPSI2 + DIPSI3 on 1H
;pl11 : f2 channel - power level for DIPSI3 on 19F
;pl13 : f1 channel - power level for NOE build up
;pl16 : f2 channel - power level for CPD/BB decoupling

;p1 : f1 channel - 90 degree 1H high power pulse
;p2 : f1 channel - 180 degree 1H high power pulse
;p3 : f2 channel -  90 degree 19F high power pulse
;p8 : Spinlock pulse [2.5 msec]
;pl8 : f2 channel - no power  [1000dB]

;d9 : CP time n*p25*217.3 = n*40*217.3 = n*8.69 ms
;d10 : DIPSI-2 spin lock time  n*p6*115.112 - n*40*115.1 = n*4.6 ms

;p44 : 180 deg BB 19F pulse
;spnam30: Crp140,1,20.1
;spdB30: power level for 180 degree 19F shape pulse

;p32: f1 channel - 180 degree shaped pulse (adiabatic)      [20
msec]
;     smoothed chirp (sweepwidth, 20% smoothing, 10000 points)
;sp29: f1 channel - shaped pulse (adiabatic)

;cpd3 : garp4
;d0 : incremented delay (2D)
;d1 : relaxation delay; 1-5 * T1
;d11: delay for disk I/O                              [30 msec]
;d12: delay for power switching                       [20 usec]
;inf1: 1/SW(H) = 2 * DW(H)
;in0: 1/(2 * SW(H)) = DW(H)
;nd0: 2
```

```
;ns: 2 * n
;ds: 4
;td1: number of experiments
;FnMODE: States-TPPI, TPPI, States or QSEQ

;Processing

;if zgoptns -DF1180
;PHC0(F1): 90
;PHC1(F1): -180
;FCOR(F1): 1

;else
;PHC0(F1): 0
;PHC1(F1): 0
;FCOR(F1): 0.5
```

Pulse program PP.4. 2D ^{19}F, ^{13}C HMBC optimised for $^{n}J_{FC}$ coupling constants

```
;19FX_nJFC_hmbcgp.as
;gradient selected, nonrefocused HMBC for correlation via nJCF or
;nJNF
;phase sensitive using Echo/Antiecho gradient selection
;1H decoupling during acquisition
;
;A.J.R. Smith, R. York, D. Uhrín and N.G.A. Bell(to be completed)
;D.O. Cicero, G. Barbato & R. Bazzo, J. Magn. Reson. 148,
;209-213 (2001)
;
;$CLASS=HighRes
;$DIM=2D
;$TYPE=
;$SUBTYPE=
;$COMMENT=


#include <Avance.incl>
#include <Grad.incl>
#include <Delay.incl>


"cnst30=(1-sfo2/sfo1)/(1+sfo2/sfo1)"

define list<gradient> EA1 = { 1.000 -cnst30}
define list<gradient> EA2 = { -cnst30 1.000}



"d6=1s/(cnst13*4)"
"d11=30m"
"d12=20u"

"in0=inf1/2"

# ifdef F1180
"d0=0.5*in0"
"DELTA=p44"
# else
"d0=3u"
"DELTA=p44+d0*2"
# endif


"DELTA3=2*p16+2*d16+p24+DELTA+8u"
"DELTA1=d6-DELTA3/2"
"DELTA2=d6-p14+p1*2/3.1416+DELTA3/2"
"acqt0=0"

1 ze
  d12 pl16:f3
2 d11 do:f3
  d12 BLKGRAD
  d1 pl1:f1 pl8:f2
```

```
3 d12 cpds2:f3
  p1 ph1
  DELTA1 pl0:f1
  (p44:sp30 ph2):f1
  4u
  (p14:sp3 ph1):f2
  4u
  DELTA2 UNBLKGRAD pl2:f2

  (p3 ph3):f2
  d0
  (p44:sp30 ph2):f1   ;middle of t1 period
  d0
  4u do:f3
  p16:gp1*EA1
  d16 pl8:f2
  (p24:sp7 ph4):f2
  4u
  p16:gp1*EA2
  d16 pl2:f2
  DELTA cpds2:f3
  (p3 ph4):f2

  go=2 ph31
  d11 do:f3 mc #0 to 2
  F1EA(calgrad(EA1) & calgrad(EA2), caldel(d0, +in0) & calph(ph3,
+180) & calph(ph31, +180))
  4u BLKGRAD

     exit


ph1=0
ph2=0 0 2 2
ph3=0 2
ph4=0 0 0 0 2 2 2 2
ph31=0 2 0 2 2 0 2 0


;pl1 : f1 channel - power level for pulse (default)
;pl2 : f2 channel - power level for pulse (default)
;pl0 : f1 channel - no power  [1000dB]
;pl8 : f2 channel - no power  [1000dB]
;pl16: f3 channel - power level for 1H CPD/BB decoupling

;p1 : f1 channel -  90 degree high power pulse
;p3 : f2 channel -  90 degree high power pulse

;sp3: f2 channel - shaped pulse (180degree inversion)
;spnam3 : Crp60,0.5,20.1
;p14: f2 channel - 180 degree shaped pulse for inversion [500usec]
;sp7: f2 channel - shaped pulse (180degree refocussing)
;spnam7: Crp60comp.4
;p24: f2 channel - 180 degree shaped pulse for refocussing [2ms]
```

```
;p44 : 180 deg BB 19F pulse
;spnam30: Crp140,1,20.1
;spdB30: power level for 180 degree 19F shape pulse

;p16: homospoil/gradient pulse                        [1 msec]
;d16: delay for homospoil/gradient recovery
;d0 : incremented delay (2D)
;d1 : relaxation delay; 1-5 * T1
;d6 : delay for evolution of long - couplings (0.25/nJFC)
;cnst13: = nJFC (nJFN)
;inf1: 1/SW(X) = 2 * DW(X)
;in0: 1/(2 * SW(X)) = DW(X)
;nd0: 2
;ns: 2 * n
;ds: 16
;td1: number of experiments
;FnMODE: echo-antiecho

;gpz1: 80%
;gpnam1: SMSQ10.100

;Processing

;if zgoptns -DF1180
;PHC0(F1): 90
;PHC1(F1): -180
;FCOR(F1): 1

;else
;PHC0(F1): 0
;PHC1(F1): 0
;FCOR(F1): 0.5
```

Pulse program PP.5. 2D ^{19}F, ^{13}C HMBC optimised for $^1J_{FC}$ coupling constants

```
;19F13C_1JFC_hmbcgp.as
;gradient selected, nonrefocused HMBC for correlation via 1JCF
;phase sensitive using Echo/Antiecho gradient selection
;1H decoupling during acquisition
;
;A.J.R. Smith, R. York, D. Uhrín and N.G.A. Bell(to be completed)
;D.O. Cicero, G. Barbato & R. Bazzo, J. Magn. Reson. 148,
;   209-213 (2001)
;
;$CLASS=HighRes
;$DIM=2D
;$TYPE=
;$SUBTYPE=
;$COMMENT=

#include <Avance.incl>
#include <Grad.incl>
#include <Delay.incl>

"cnst30=(1-sfo2/sfo1)/(1+sfo2/sfo1)"

define list<gradient> EA1 = { 1.000 -cnst30}
define list<gradient> EA2 = { -cnst30 1.000}


"d6=1s/(cnst13*4)"
"d11=30m"
"d12=20u"

"in0=inf1/2"

# ifdef F1180
"d0=0.5*in0"
"DELTA=p44"
# else
"d0=3u"
"DELTA=p44+d0*2"
# endif


"DELTA3=2*p16+2*d16+p24+DELTA+8u"
"DELTA1=(DELTA3-d6-p14)/2+(2/3.1416)*p1+d12"
"DELTA2=(DELTA3-p14+d6)"


"acqt0=0"


1 ze
  d12 pl16:f3
2 d11 do:f3
  d12 BLKGRAD
  d1 pl1:f1 pl8:f2
```

```
3 d12 cpd2:f3
  p1 ph1
  d12 pl0:f1
  (p44:sp30 ph2):f1
  DELTA1
  (p14:sp3 ph1):f2
  DELTA2 pl2:f2 UNBLKGRAD

  (p3 ph3):f2
  d0
  (p44:sp30 ph2):f1
  d0
  4u do:f3
  p16:gp1*EA1
  d16 pl8:f2
  (p24:sp7 ph4):f2
  4u
  p16:gp1*EA2
  d16 pl2:f2
  DELTA cpd2:f3
  (p3 ph4):f2

  go=2 ph31
  d11 do:f3 mc #0 to 2
  F1EA(calgrad(EA1) & calgrad(EA2), caldel(d0, +in0) & calph(ph3,
+180) & calph(ph31, +180))
  4u BLKGRAD
     exit


ph1=0
ph2=0 0 2 2
ph3=0 2
ph4=0 0 0 0 2 2 2 2
ph31=0 2 0 2 2 0 2 0


;pl1 : f1 channel - power level for pulse (default)
;pl2 : f2 channel - power level for pulse (default)
;pl0 : f1 channel - no power  [1000dB]
;pl8 : f2 channel - no power  [1000dB]
;pl16: f3 channel - power level for 1H CPD/BB decoupling
;p1 : f1 channel -  90 degree high power pulse
;p3 : f2 channel -  90 degree high power pulse
;sp3: f2 channel - shaped pulse (180degree inversion)
;spnam3 : Crp60,0.5,20.1
;p14: f2 channel - 180 degree shaped pulse for inversion [500usec]
;sp7: f2 channel - shaped pulse (180degree refocussing)
;spnam7: Crp60comp.4
;p24: f2 channel - 180 degree shaped pulse for refocussing [2ms]
;p44 : 180 deg BB 19F pulse
;spnam30: Crp140,1,20.1
;spdB30: power level for 180 degree 19F shape pulse
;p16: homospoil/gradient pulse                        [1 msec]
;d16: delay for homospoil/gradient recovery
;d0 : incremented delay (2D)
```

```
;d1 : relaxation delay; 1-5 * T1
;d6 : delay for evolution of one-bond couplings (0.25/1JFC)
;cnst13: = 1JFC
;inf1: 1/SW(X) = 2 * DW(X)
;in0: 1/(2 * SW(X)) = DW(X)
;nd0: 2
;ns: 2 * n
;ds: 16
;td1: number of experiments
;FnMODE: echo-antiecho

;gpz1: 80%
;gpnam1: SMSQ10.100

;Processing

;if zgoptns -DF1180
;PHC0(F1): 90
;PHC1(F1): -180
;FCOR(F1): 1

;else
;PHC0(F1): 0
;PHC1(F1): 0
;FCOR(F1): 0.5
```

Pulse program PP.6. (3, 2)D $H^{1}C^{n}F$

```
;1H13C19F_2D.ry
;Reduced dimensionality interleaved HCF correlation with 13C
;chemical shifts coded as inphase and anti-phase doublets in the F1
;dimension of 2D 1H,19F correlated spectra. Gradient selected, with
;1H decoupling during acquisition yielding anti-phase nJCF doublets
;in F2.

;A.J.R. Smith, R. York, D. Uhrín and N.G.A. Bell(to be completed)


#include <Avance.incl>
#include <Grad.incl>
#include <Delay.incl>


"p22=p21*2"
"d11=30m"
"d12=20u"

"d2=0.25/cnst10"            ;cnst10 = 1J(13C-1H)
"d3=0.5/cnst10"
"d4=0.25/cnst11"            ;cnst11 = nJ(13C-19F)
"d6=cnst1/cnst10"           ;cnst1 = 0.5 for CH, 0.25 for CH2
"d5=d4-d6"
"DELTA1=d3-p14/2"
"DELTA2=d3-p14/2-p44"
"DELTA3=d2-p16-d16-p14/2"
"DELTA4=d2+d0*2-p14/2"
"DELTA5=p16+d16+4u-p1*2/3.1416"

"d0=3u"
"in0=inf1/2"
"in33=cnst5*in0"
"in43=in33"
"d33=d5-(cnst17*p24/2)-p44"
"d43=d4-cnst17*p24/2"
"acqt0=0"

"l0=0"

baseopt_echo

1 ze
2 d11
  d12 do:f3
  d12 pl1:f1 pl3:f3
  d12 BLKGRAD
  d1
  d12 UNBLKGRAD
  p1 ph1
  2u
  p1 ph3
  p16:gp3
  d16
```

```
   (p21 ph4):f3   ;1H 90 degree pulse channel 3

   d0
   (p21 ph1):f3           ;1H 90 degree pulse channel 3 BIRDrx
   DELTA1 pl8:f2 pl0:f1
   (center (p22 ph3):f3 (p14:sp3 ph1):f2 )   ;180 degree pulse
   (p44:sp30 ph1):f1
   DELTA2
   (p21 ph1):f3  ;1H 90 degree pulse on channel 3, end of BIRD
   d0

   DELTA3
   p16:gp1*EA
   d16
   (center (p22 ph1):f3 (p14:sp3 ph1):f2 )
   DELTA4 pl2:f2
   (p21 ph2):f3        ;transfer to 13C
   (p3 ph7):f2
   d6 pl16:f3          ;pl16 decoupling power channel 3
   d33 pl8:f2 cpd2:f3  ;d33 is incrementing t2
   4u
   (p44:sp30 ph1):f1
   (p24:sp7 ph1):f2
   4u
   d43 pl2:f2

 if "l0 %2 == 0"
 {
      (p3 ph5):f2    ;cos
 }
 else
 {
      (p3 ph6):f2    ;sin
 }

   d12 do:f3 pl1:f1
   p1 ph1
   DELTA5 pl0:f1
   (p44:sp30 ph1):f1
   4u
   p16:gp2
   d16
   (p44:sp30 ph1):f1
   go=2 ph31 cpd2:f3
   d11 do:f3 mc #0 to 2
  F1I(iu0,2)

  F1EA(calgrad(EA),caldel(d0,+in0) & caldel(d33, +in33) & caldel(d43,
 -in43) & calph(ph4, +180) & calph(ph31, +180))
  d12 BLKGRAD
  exit

 ph1 = 0
 ph2 = 2
 ph3 = 1
```

```
ph4 = 1 3
ph5 = 0 0 2 2
ph6 = 1 1 3 3
ph7 = 0 0 0 0 2 2 2 2
ph31 = 0 2 2 0 2 0 0 2

;pl1 : f1 channel - 1H power level for pulse (default)
;pl2 : f2 channel - 13C power level for pulse (default)
;pl3 : f3 channel - 19F power level for pulse (default)
;pl0 : 0W decoupling power on channel 1
;pl8 : 0W decoupling power on channel 2
;pl16 decoupling power on channel 3 1H
;p1 : f1 channel - 19F 90 degree high power pulse
;p3 : f2 channel - 13C 90 degree high power pulse
;p21: f3 channel - 1H 90 degree high power pulse
;p22: f3 channel - 1H 90 degree high power pulse
;sp3: f2 channel - shaped pulse (180degree inversion)
;spnam3 : Crp60,0.5,20.1
;p14: f2 channel-180 degree shaped inversion pulse [500usec]
;sp7: f2 channel - shaped pulse (180degree refocussing)
;spnam7: Crp60comp.4
;p24: f2 channel-180 degree shaped pulse for refocussing [2ms]
;p44 : 180 deg BB 19F pulse
;spnam30: Crp140,1,20.1
;spdB30: power level for 180 degree 19F shape pulse
;d0 : incremented delay (2D)     [3 usec]
;d1 : relaxation delay; 1-5 * T1
;d11: delay for disk I/O    [30 msec]
;d12: delay for power switching  [20 usec]
;cnst1 : 0.5 for CH, 0.25 for all multiplicities
;cnst5 ; scaling factor for the evolution of 13C chemical shift
;cnst10 : 1JCH coupling 13C-1H
;cnst11 : J coupling 13C-19F
;cnst17 = -0.5 for Crp60comp.4
;inf1: 1/SW(H) = 2 * DW(H)
;in0: 1/(2 * SW(H)) = DW(H)
;nd0: 2
;ns: 2 * n
;ds: 16
;td1: number of experiments
;FnMODE: States-TPPI, TPPI, States or QSEQ
;use gradient ratio: gp1 : gp2
;                     40 : 42.52 for F-19
```
